# Three new species of the family Canuellidae Lang, 1944 (Copepoda, Canuelloida) from the Western Pacific Ocean

**DOI:** 10.3897/zookeys.1273.176856

**Published:** 2026-03-23

**Authors:** Hyun Woo Bang, Jinwook Back

**Affiliations:** 1 Division of Biomedical Engineering and Biotechnology, Mokwon University, Daejeon, 35349, Republic of Korea Division of Biomedical Engineering and Biotechnology, Mokwon University Daejeon Republic of Korea; 2 Department of Taxonomy and Systematics, National Marine Biodiversity Institute of Korea, Seocheon, 33662, Republic of Korea Department of Taxonomy and Systematics, National Marine Biodiversity Institute of Korea Seocheon Republic of Korea

**Keywords:** 18S rRNA, *
Brianola
*, *
Elanella
*, Korea, molecular phylogeny, mtCOI, Palau, *
Scottolana
*

## Abstract

Three new species of the copepod family Canuellidae Lang, 1944 (Copepoda, Canuelloida) are described from the Western Pacific Ocean, based on specimens collected from coastal waters of Korea (Jeju-do Island) and the Republic of Palau. This study provides detailed morphological descriptions and illustrations for *Elanella jejuensis***sp. nov**., *Brianola coreana***sp. nov**., and *Scottolana picrca***sp. nov**. Key diagnostic characters differentiating them from congeners include the unique bulbous modification of caudal ramal seta IV in *E. jejuensis***sp. nov**., the presence of an inner seta on the P1 exopod-2 in *B. coreana***sp. nov**., and features including reduced urosome segmentation and a modified female caudal seta II that place *S. picrca***sp. nov**. within the *longipes*-group. Molecular phylogenetic analysis using mitochondrial cytochrome *c* oxidase subunit I (COI) and nuclear 18S ribosomal RNA (18S rRNA) sequences recovers each new species as a distinct, well-supported lineage and resolves two major clades within Canuellidae: (*Elanella* + *Brianola*) and (*Scottolana* + *Canuella*). The phylogeny further indicates that *Elanella* and *Brianola* are sister taxa, and places *S. picrca***sp. nov**. within the *longipes*-group; however, the placement of *S. picrca***sp. nov**. as sister to *S. wonchoeli* is weakly supported (57% bootstrap). These findings significantly advance our understanding of Canuellidae diversity and evolution in the understudied Western Pacific region.

## Introduction

The taxonomic position of the families Canuellidae Lang, 1944 and Longipediidae Boeck, 1865, both currently classified within the order Canuelloida, has undergone significant revision. Lang (1948), who revised the phylogenetic relationships among the copepod families of Harpacticoida, originally grouped these families into the section Polyarthra, differentiating them from other harpacticoids (Oligoarthra) based on distinct morphological traits observed in adult and naupliar stages (Gómez et al. 2024). However, the monophyly of Harpacticoida, as defined by Lang, was later challenged (Tiemann 1984). Subsequent comparative studies, particularly those emphasizing the absence of shared derived naupliar characteristics and distinct patterns in naupliar development between Polyarthra and Oligoarthra (Dahms 1990, 2004a, 2004b), strongly suggested that Polyarthra represents a distinct monophyletic lineage separate from the core harpacticoids (Huys et al. 1996; Gómez et al. 2024). Based largely on this naupliar evidence, Dahms (2004a) ultimately removed Polyarthra from Harpacticoida.

Molecular studies have further refined this view. On the basis of molecular phylogenetic analyses, Khodami et al. (2017) proposed the ordinal name Canuelloida, confirming its separation from Harpacticoida*sensu stricto* (Oligoarthra). Although this paper was later retracted (Khodami et al. 2020), which would typically render nomenclatural acts unavailable under the standard provisions of the International Code of Zoological Nomenclature (ICZN 1999), the name Canuelloida remains available. This availability follows Declaration 46 (ICZN 2023), which amends Article 8.8 to state that retraction does not affect the availability of names therein (see discussion in Gómez et al. 2024). Recent phylogenomic analyses strongly support Canuelloida as a valid order distinct from Harpacticoida (Bernot et al. 2025). In light of these morphological, molecular, and nomenclatural discussions, the present study adopts the order Canuelloida as encompassing the families Canuellidae and Longipediidae. While the family Longipediidae consists of a single monotypic genus, the family Canuellidae currently comprises at least 67 species in 18 genera; the World of Copepods Database lists 66 species (Walter and Boxshall 2026), but this total has not yet incorporated *Brianola rawaiensis*, described by Chullasorn et al. (2024).

Canuellidae is considered to retain relatively plesiomorphic morphological characteristics within copepods (Drumm et al. 2015), although some studies have questioned its strict monophyly (Por 1984; Drumm et al. 2015). Species belonging to this family are predominantly free-living in marine sediment environments, especially in intertidal or shallow subtidal zones, with some exhibiting symbiotic relationships with other invertebrates (Boxshall and Halsey 2004; Nazari et al. 2018).

Research on the Canuellidae fauna in the Western Pacific, particularly in Korean and Palauan waters—the focal regions of this study—remains insufficient. While studies have been conducted on the Canuellidae elsewhere in Asian regions such as China (Mu and Huys 2004), Korea (Bang et al. 2022), and Thailand (Song et al. 2018; Chullasorn et al. 2024), the full diversity in these areas has yet to be elucidated. In recent meiobenthic surveys of coastal waters of Korea and Palau, specimens referable to the canuellid genera *Scottolana* Huys, 2009, *Elanella* Por, 1984, and *Brianola* Monard, 1926 were collected. Among these, *Scottolana* is currently the most speciose genus within Canuellidae, with 20 valid species, while *Elanella* and *Brianola* include three and nine species, respectively (Drumm et al. 2015; Chullasorn et al. 2024; Gómez et al. 2024). However, records of *Scottolana* in Asian marine waters remain scarce (Mu and Huys 2004; Song et al. 2018; Bang et al. 2022).

Meiobenthos is an important component of marine ecosystems, contributing to energy flow and nutrient cycling; documenting meiobenthic biodiversity and community structure is essential for understanding marine ecosystem structure and function (Coull 1999; Giere 2009). Meiobenthic copepods, in particular, respond sensitively to environmental changes and hold potential as bioindicators reflecting specific environmental conditions (Hicks and Coull 1983; Heip et al. 1985; Coull and Chandler 1992; Gómez et al. 2024).

This study aims to address knowledge gaps regarding Canuellidae diversity in the Western Pacific by providing new morphological and molecular data that will contribute to the systematics and ecological understanding of this family. Herein, we provide detailed morphological descriptions of three new canuellid species: two collected from Korean waters and one from Palauan waters. Furthermore, DNA barcode sequences of the mitochondrial cytochrome c oxidase subunit I (COI) gene and the 18S ribosomal RNA gene are presented for the two new species discovered in Korea.

## Materials and methods

In the Republic of Palau, the collection of harpacticoids was carried out under a Palau government permit (RE-19-09) and a Koror state government permit (T19-10750, Permit No. 048). Samples were collected using a hand net, grab sampler, light trap, or by SCUBA diving, and then fixed in 95% ethanol (Table 1). Canuellid copepods were sorted from the samples with the aid of a Leica M80 stereomicroscope. The sorted specimens were stored at –20 °C before DNA extraction. All specimens were morphologically observed following non-destructive DNA extraction (Vakati and Dodsworth 2020).

**Table 1.** Information of collection and specimens in this study.

Species name	Collect dates	GPS coordinates	Method (depth)	Specimen Nos.
*Elanella jejuensis* sp. nov.	13-Jun.-2017	33°13'51.83"N, 126°13'47.45"E	SCUBA (10 m)	MABIK CR00259503
				MABIK CR00259504
	03-Jul.-2019	33°29'51.36"N, 126°55'6.42"E	Grab (20 m)	MABIK CR00259505
				MABIK CR00259506
				MABIK CR00259507
*Brianola coreana* sp. nov.	14-Mar.-2017	33°32'21.95"N, 126°50'17.45"E	Hand net (1 m)	MABIK CR00242447
				MABIK CR00242448
				MABIK CR00242449
				MABIK CR00242450
				MABIK CR00242451
	15-Mar.-2017	33°32'21.95"N, 126°50'17.45"E	Hand net (1 m)	MABIK CR00242532
				MABIK CR00242533
				MABIK CR00242534
*Scottolana picrca* sp. nov.	21-Jan.-2019	7°19'47.91"N, 134°27'20.43"E	Light trap (5 m)	MABIK CR00259508
				MABIK CR00259509
				MABIK CR00259510
				MABIK CR00259511
				MABIK CR00259512

Specimens were dissected in lactic acid and mounted on slides using lactophenol as a mounting medium. Transparent nail varnish was used to seal the preparations. All drawings were created on a differential interference contrast microscope (Leica DM 2500) with a camera lucida. The descriptive terminology regarding the body and appendage morphology follows Huys and Boxshall (1991).

Abbreviations used in the text are: **A1** for antennule; **A2** for antenna; ae for aesthetasc; **exp** for exopod; **enp** for endopod; **P1–P6** for the first to the sixth thoracopods; **exp (enp)-1 (-2, -3)** to denote the proximal (middle, distal) segments of a ramus. All specimens were deposited in the National Marine Biodiversity Institute of Korea (**MABIK**; Seocheon, Republic of Korea). Accession numbers are provided in Table 2 alongside each specimen.

**Table 2.** GenBank number of sequences used in phylogenetic analyses.

Family	Species name	Accession no.			
		18SrDNA	Specimen No.	mtCOI	Specimen No.
Canuellidae	*Brianola coreana* sp. nov.	PP298071	MABIK CR00242533	PP297485	MABIK CR00242533
	*Canuella perplexa* Scott T. & Scott A., 1893	MF077726	–	MH670545	–
	*Elanella jejuensis* sp. nov.	PP298074	MABIK CR00259507	PP297487	MABIK CR00259506
	*Scottolana daecheonensis* Bang, Moon & Back, 2022	OP454042	MABIK CR00252813	OP452901	MABIK CR00252813
	*Scottolana picrca* sp. nov.	PP298076	MABIK CR00259511	PP297490	MABIK CR00259511
	*Scottolana wonchoeli* Bang, Moon & Back, 2022	OP454040	MABIK CR00260952	PP297495	MABIK CR00260952
Longipediidae	*Longipedia coronata* Claus, 1863	PP809124	–	MH976656	–
	*Longipedia koreana* Bang, Moon & Back, 2021	OK335989	MABIK CR00248539	OK333366	MABIK CR00248539
	*Longipedia ulleungensis* Bang, Moon & Back, 2021	OK339013	MABIK CR00248543	OK333377	MABIK CR00248543

DNA extraction was conducted following the manufacturer’s protocol for the Qiagen DNeasy Blood and Tissue Kit (Qiagen, Hilden, Germany). PCR amplification of nuclear 18S ribosomal RNA (18S rRNA) and mitochondrial cytochrome *c* oxidase subunit I (mtCOI) genes was performed using the AccuPower HotStart PCR PreMix (Bioneer, Daejeon, Korea). General procedures for DNA extraction and PCR followed Lim et al. (2020). The PCR products were sequenced using an ABI PRISM 3730XL Analyzer (Macrogen Inc., Seoul, Korea) in both directions. Geneious Prime 2025.2.2 (Biomatters, Auckland, New Zealand) was used to assemble the sequences (Kearse et al. 2012). Phylogenetic analyses were conducted using concatenated mtCOI and 18S rRNA sequences from four genera of Canuellidae. Sequences of three Longipediidae species were downloaded from GenBank; accession numbers are also listed in Table 2. The alignments of each gene set were constructed using MAFFT version 7.520 (Katoh and Standley 2013) with the L-INS-i algorithm. Phylogenetic relationships were inferred by Maximum Likelihood (ML) under the General Time Reversible (GTR) with a gamma-distributed rate variation among sites (+G) and a proportion of invariable sites (+I) (Kimura 1980; Nei and Kumar 2000), as determined by model selection in MEGA X. Node support values were assessed using 1,000 bootstrap replicates.

## Systematics

### Order Canuelloida Khodami, Vaun MacArthur, Blanco-Bercial & Martínez Arbizu, 2017

**Family Canuellidae Lang, 1944**

**Genus *Elanella* Por, 1984**

#### Elanella jejuensis sp. nov.

Taxon classificationAnimaliaCanuelloidaCanuellidae

1917DBA7-1FFF-5CAD-8E4C-27436BA2DCA4

https://zoobank.org/6A7A4D17-46B6-4921-97DE-7552DE9BB75E

Figs 1, 2, 3, 4, 5, 6, 7, 8, 9, 10, 11

##### Type locality.

A subtidal zone near Dongil-ri Port in Jeju-do Island, Korea (33°13'52"N, 126°13'47"E), 10 m depth.

##### Type material.

***Holotype*** • 1♀ (MABIK CR00259503) dissected on fifteen slides. ***Paratypes*** • 1♂ (MABIK CR00259504) dissected on ten slides, and • 2♀♀ (MABIK CR00259505, MABIK CR00259507) and • 1♂ (MABIK CR00259506) preserved in 99% alcohol.

##### GenBank accession numbers.

Mitochondrial cytochrome c oxidase subunit I gene (PP297487, PP297488); 18S ribonucleic acid gene (PP298073, PP298074).

##### Description of female.

**Figure 1.**
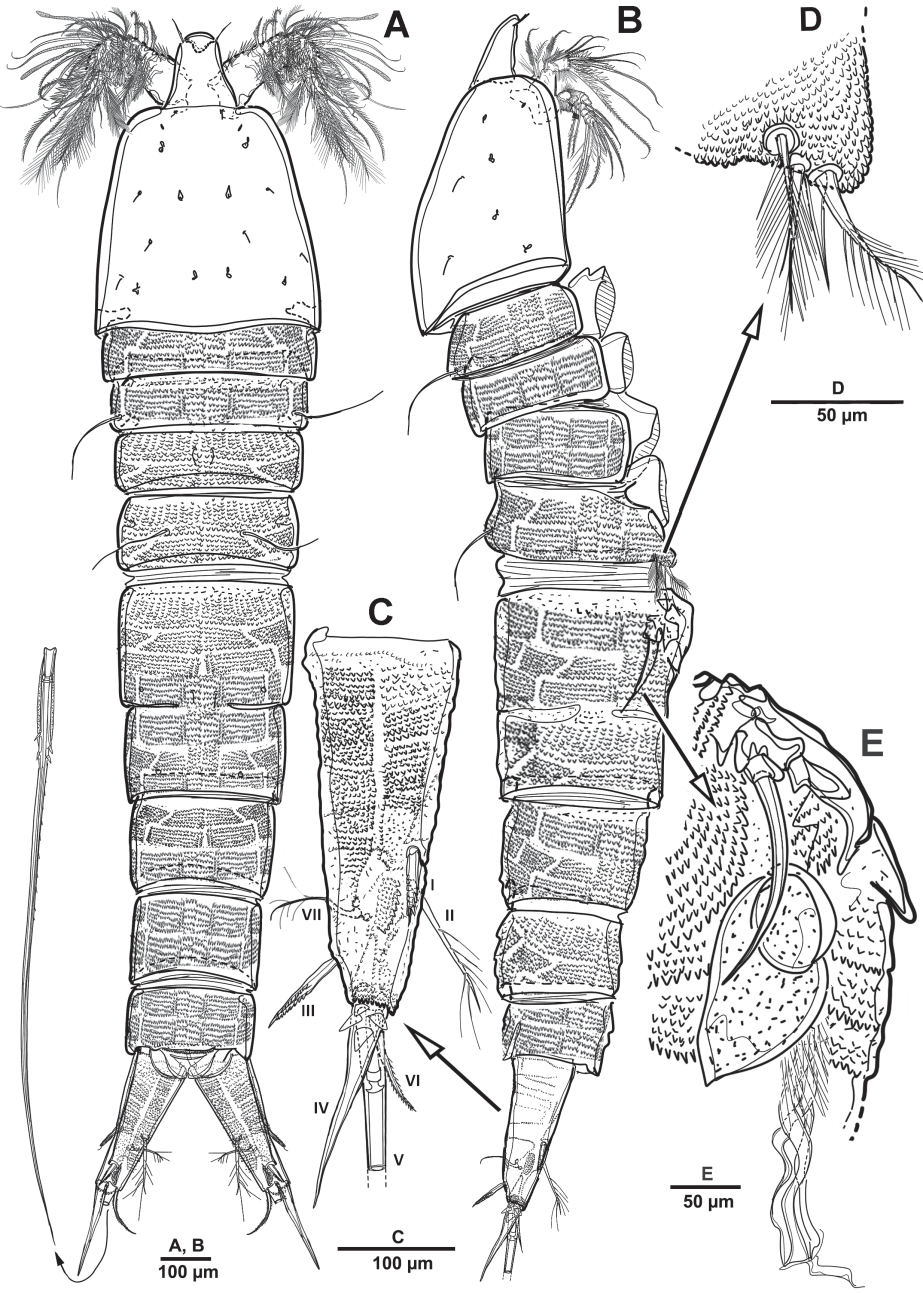
*Elanella jejuensis* sp. nov., holotype female. **A**. Habitus, dorsal; **B**. Habitus, lateral; **C**. Caudal ramus, lateral; **D**. P5; E. P6.

Body (Fig. 1A, B) length 2,240 µm (measured from tip of rostrum to posterior margin of caudal rami). Largest width measured at posterior margin of cephalic shield: 448 µm. Body semi-cylindrical, and surface covered with tiny denticles.

Prosome (Fig. 1A, B) consisting of cephalosome and four distinct pedigerous somites. First pedigerous somite completely separated from cephalosome.

**Figure 2.**
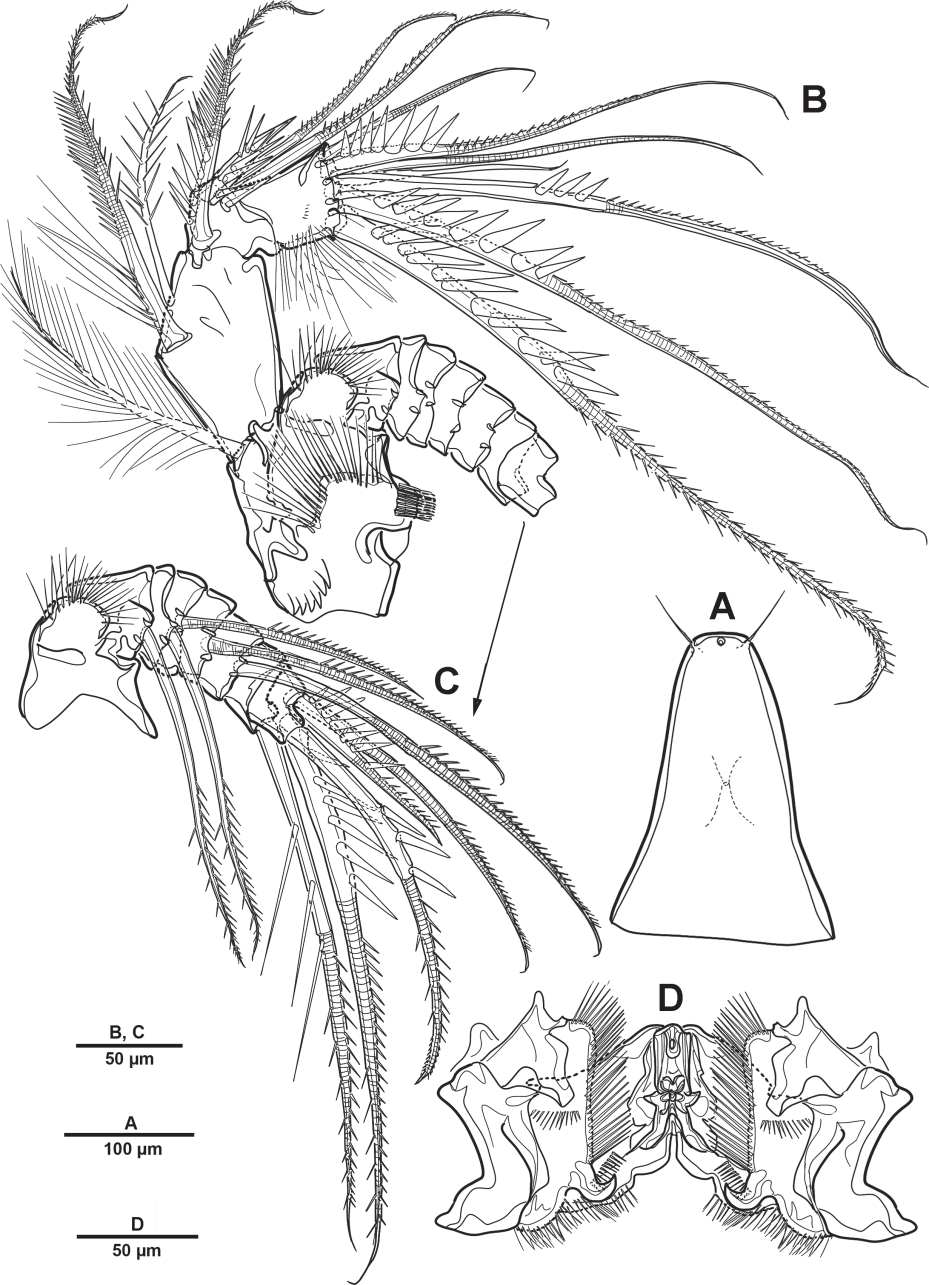
*Elanella jejuensis* sp. nov., holotype female. **A**. Rostrum; **B**. Antenna; **C**. Antennal exopod; **D**. Paragnaths.

Rostrum (Fig. 2A) well-developed and bell-shaped, defined at base, with pair of sensilla near anterior margin, median integumental pore, and apical pore.

**Figure 3.**
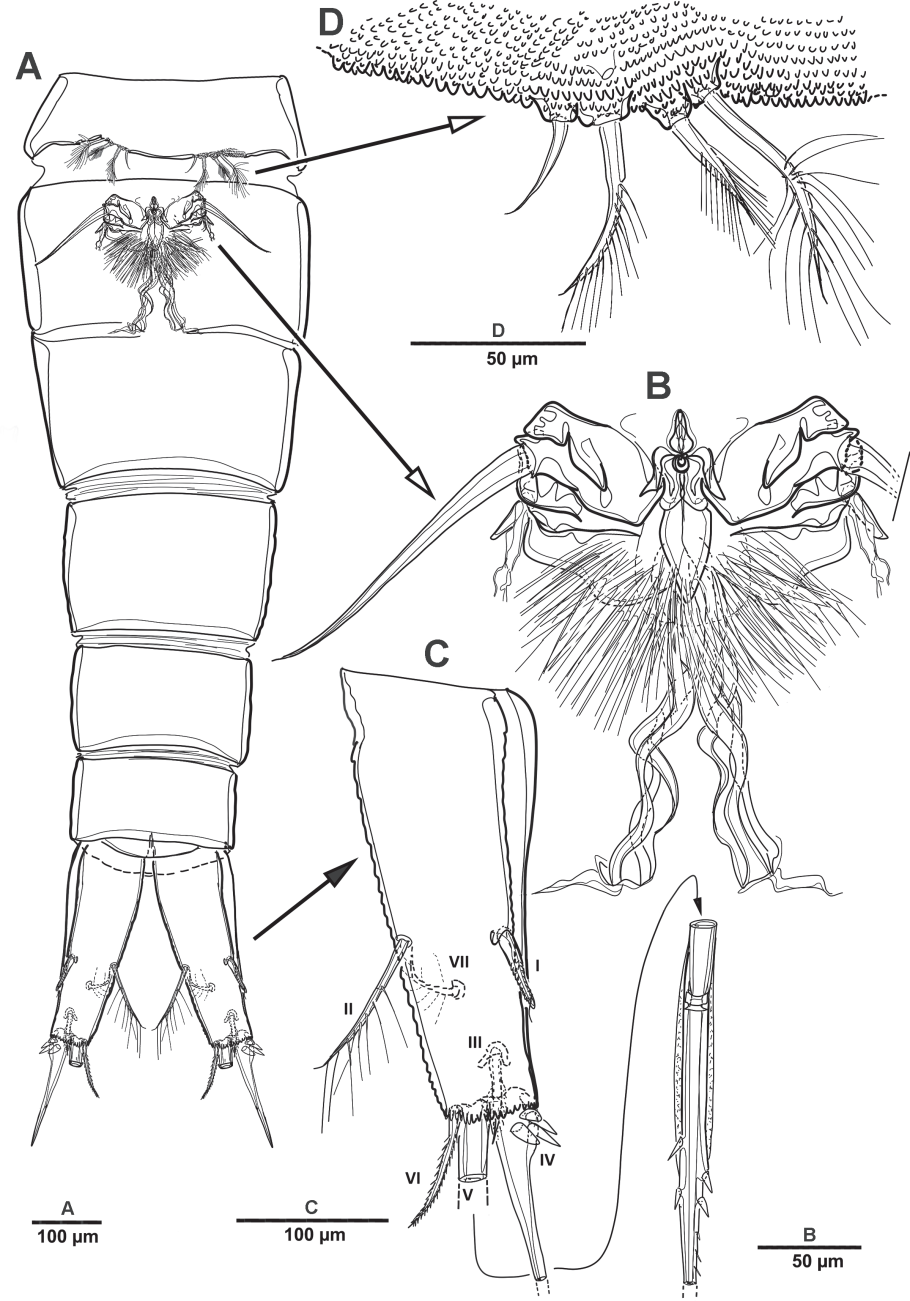
*Elanella jejuensis* sp. nov., holotype female. **A**. Urosome, ventral; **B**. Genital field and P6; **C**. Caudal ramus, ventral; **D**. P5.

Urosome (Figs 1A, 3A) slightly narrower than prosome, tapering posteriorly, consisting of P5-bearing somite, genital double-somite, and three abdominal somites, hyaline posterior fringes of urosomites plain. Genital double somite marked by internal, transverse chitinous ribs laterodorsally and laterally (Fig. 3A). Genital field (Fig. 3B) positioned near anterior margin on mid-ventral side of genital double-somite, with mid-ventral patch of long setules. Anal somite (Figs 1A, 3A) covered with tiny denticles, anal operculum weakly developed.

Caudal rami (Figs 1C, 3C) 2.4 times as long as wide, with serrate distal margin, each ramus with seven setae; seta I spiniform, serrate and short; seta II plumose, inserted halfway on inner margin; seta III spiniform and short, displaced to dorsal surface; seta IV–VI displaced on distal margin; seta IV bulbous, basally spinulate; seta V longest, longer than all urosomites combined; seta VI short and bipinnate; seta VII short and smooth, bi-articulate at base.

**Figure 4.**
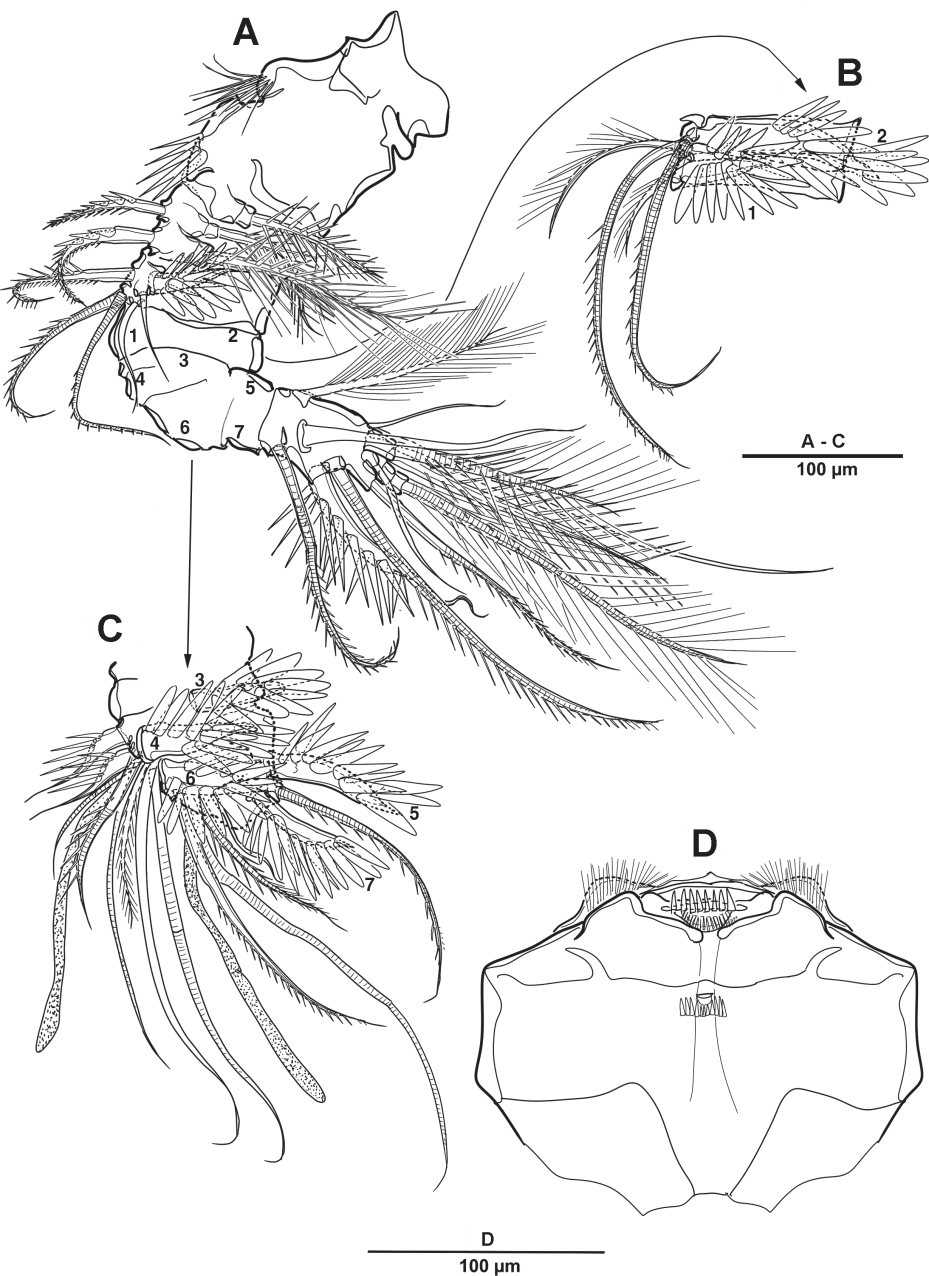
*Elanella jejuensis* sp. nov., holotype female. **A**. Antennule; **B**. Antennular segment 2; **C**. Antennular segment 3; **D**. Labrum. Numerals (1–7) indicate strong pinnate spine-like setae.

Antennule (Fig. 4A–C) 4-segmented. First segment largest, featuring incomplete transverse sutures along posterior margin and row of fine setules and row of spinules along anterior margin; with three strong pinnate, three tri-articulate pinnate, one strong spine-like, two geniculate, and two naked setae. Segment 2 (Fig. 4B) short, with two unipinnate, two geniculate, and two strong pinnate spine-like setae (numbered 1 and 2 in Fig. 4A, B). Segment 3 (Fig. 4C) with four pinnate, five strong spine-like (numbered 3–7 in Fig. 4A, C), seven geniculated, and two naked setae, and two aesthetascs. Distal segment with two plumose, one strong spine-like, four naked, and five geniculated setae (two of which are bi-articulate and one is tri-articulate). Armature as follows: 1-[11], 2-[6], 3-[18 + 2 ae]. 4-[12].

Antenna (Fig. 2B, C) biramous, composed of coxo-basis, exp, and enp. Coxo-basis with one plumose seta and long setules along inner margin, and two rows of setules on anterior surface. Exp 7-segmented; first to sixth exopodal segments each with plumose/pinnate seta, distal segment with four pinnate setae. Enp 3-segmented; enp-1 with two pinnate setae; enp-2 small, with four pinnate elements; enp-3 with four pinnate and three bare setae.

Labrum (Fig. 4D) well-developed, large proximal pore surrounded by row of spinules, subdistally with row of stout spinules and row of delicate spinules, with fine setules along distal margin. Paragnaths (Fig. 2D) well-developed lobes, bearing one complex arrangement of long setules as illustrated.

**Figure 5.**
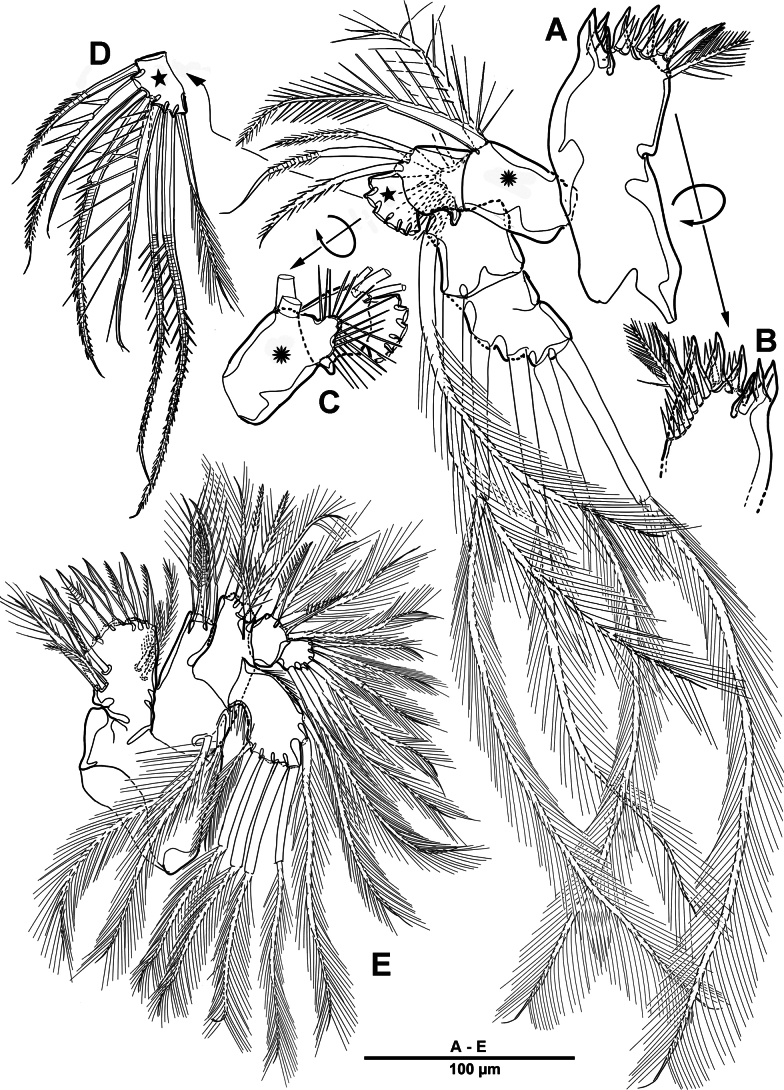
*Elanella jejuensis* sp. nov., holotype female. **A**. Mandible; **B**. Mandibular gnathobase; **C**. Mandibular basis; **D**. Mandibular enp-2; **E**. Maxillule.

Mandible (Fig. 5A–D), gnathobase (Fig. 5B) with five strong teeth and several stout spinules; dorsal corner with one plumose seta and one serrated spine. Basis (Fig. 5C) with prominent process bearing two rows of spinules and two pinnate inner setae. Enp 2-segmented; enp-1 with three inner pinnate setae; enp-2 (Fig. 5D) with six pinnate, one bare, and one plumose setae. Exp 2-segmented, all setae plumose; exp-1 with one proximal and one subdistal setae on lateral margin; exp-2 with incomplete suture, with four setae.

Maxillule (Fig. 5E). Praecoxa and coxa incompletely fused. Praecoxal arthrite with eight elements around distal margin, three elements near dorsal margin, and two setae on anterior surface. Coxa with cylindrical endite bearing two plumose setae and one pinnate spine; epipodite represented by two plumose setae. Basis with five plumose setae. Exp slightly bent outwards, 1-segmented; with two inner, four distal, and one outer small setae; all setae plumose. Enp shorter than exp, 2-segmented; enp-1 with three plumose setae and two pinnate spines; enp-2 with six plumose setae.

**Figure 6.**
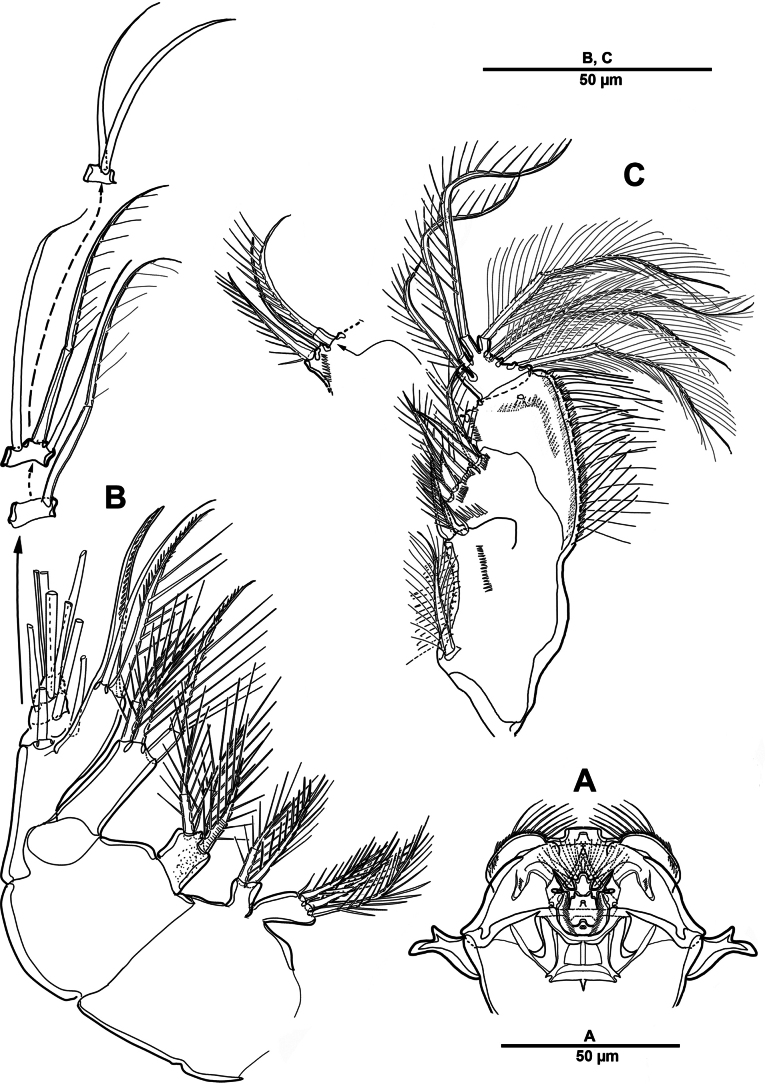
*Elanella jejuensis* sp. nov., holotype female. **A**. Maxilla; **B**. Maxillary allobasis and endopod; **C**. Maxilliped; **D**. Maxillipedal syncoxa.

Maxilla (Fig. 6A, B). Praecoxa and coxa separated by a complete suture, each with two endites; proximal endite with three pinnate setae; second endite with one pinnate seta; third endite with three setae; distal endite with three pinnate elements. Allobasis produced into one strong bare claw, with one strong unipinnate seta, two strong pinnate spines, and two bare setae. Enp seemingly 1-segmented; with seven bare setae.

Maxilliped (Fig. 6C, D) phyllopodial. Syncoxa (Fig. 6D) with row of spinules on surface and inner margin with row of long setules; with one praecoxal seta, and eight setae/spine on inner margin. Basis with row of long outer spinules; with one unipennate and one bare setae. Enp 1-segmented, with six inner pinnate and four outer plumose setae.

**Figure 7.**
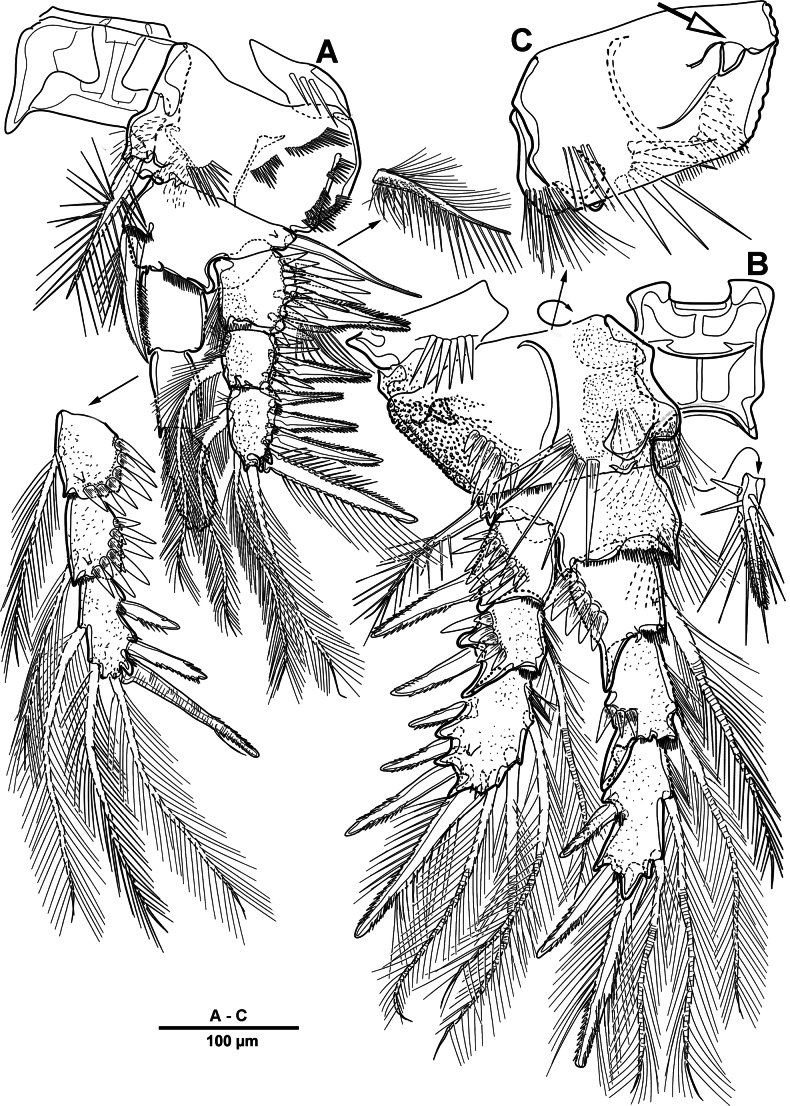
*Elanella jejuensis* sp. nov., holotype female. **A**. P1; **B**. P2; **C**. P2 coxa, posterior detail.

**Figure 8.**
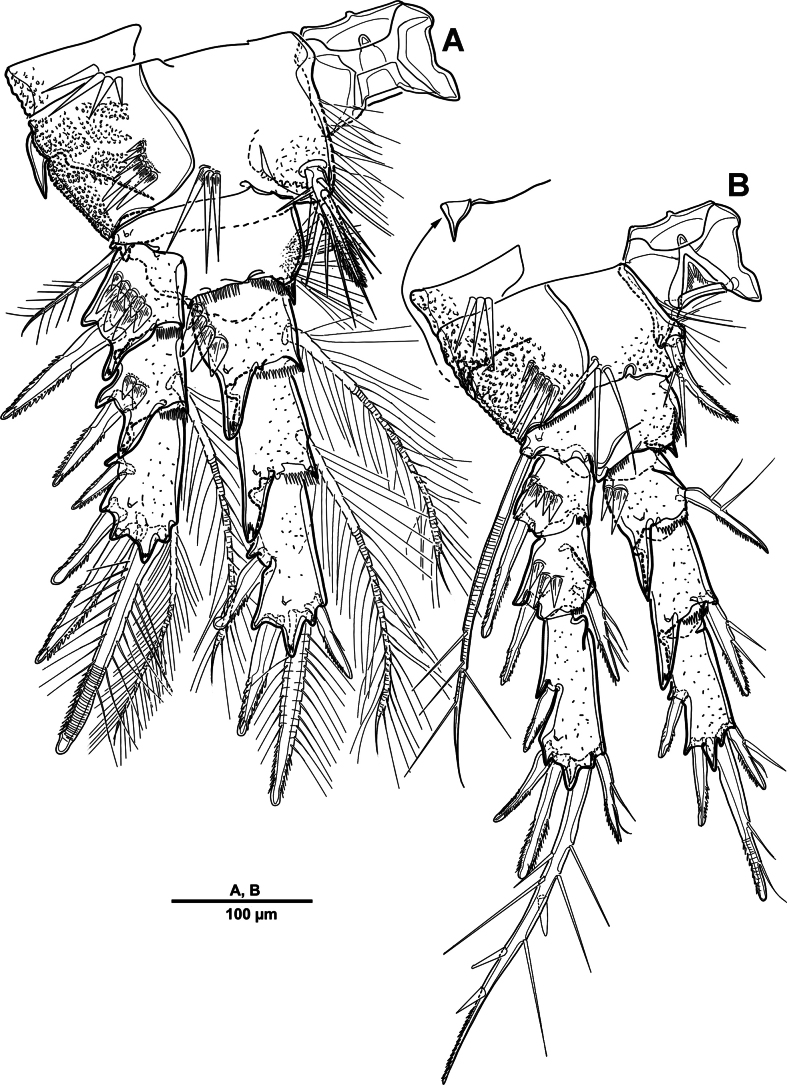
*Elanella jejuensis* sp. nov., holotype female. **A**. P3; **B**. P4.

Swimming legs 1–4 (Figs 7, 8) biramous, with well-developed praecoxae and 3-segmented rami; with intercoxal sclerites large and square. Coxae and bases with rows of spinules on anterior surface as ﬁgured.

P1 (Fig. 7A) smaller than other swimming legs. Coxa large and rectangular, with anterior patches of spinules and rows of setules on posterior surface; with bipinnate strong inner seta. Basis with one tripinnate outer seta, one strong pinnate inner spine, and one tube pore near outer setophore. Exp 3-segmented; exp-1 with one strong serrate outer spine, outer margin with strong spinules, and inner margin with setules; exp-2 with one plumose inner seta and one outer strong pinnate spine, ornamentation as previous segment; exp-3 with two plumose inner setae, distal margin with two elements (one outer distal spine inclined outwardly and one distal seta), outer margin with three pinnate spines. Enp 3-segmented, as long as exp; enp-1 and enp-2 each with one inner plumose seta and spinular ornamentation as shown; enp-3 with two long plumose inner setae, two distal elements (one plumose seta and one strong spine), and two outer serrate spines.

P2 (Fig. 7B, C). Coxa large and rhombic, with two patches of spinules on anterior surface, and one inner strong pinnate spine; posterior surface with small spinous process (arrowed in Fig. 7C). Basis with one bipinnate outer seta, process on inner distal corner, and row of spinules at base of enp. Exp-1 with one serrate outer spine, with thick spinules around outer and distal margins; exp-2 with one plumose inner seta and one serrate outer spine, outer margin with robust spinules as in exp-1; exp-3 with three long plumose inner setae, one distal spine, three outer spines, and tube pore on anterior surface at about two-thirds of segment length. Enp slightly longer than exp; enp-1 and enp-2 each with one plumose inner seta and outer distal corner produced into process, with strong spinules along outer margin; enp-3 with two plumose inner setae, one distal spine, and two outer spines.

P3 (Fig. 8A). Coxa large, with spinular ornamentation as figured, one pinnate inner seta, and outer corner produced into small process. Basis similar to P2. Exp-1 with one serrate spine and strong spinules on anterior surface; exp-2 with one plumose inner seta and one outer spine; exp-3 with one plumose inner seta, one strong apical spine, three outer spines, and tube pore on anterior surface. Enp longer than exp; enp-1–enp-3 each with tube pore on anterior surface; enp-1 and enp-2 each with one plumose inner seta and outer distal corner produced into a process; enp-3 with two terminal elements (one small serrate spine and one bipinnate spine) and two bi-serrate outer spines.

P4 (Fig. 8B). Coxa with spinular ornamentation and process similar to that of P3, with one inner seta. Basis with one long pinnate outer seta. Exp-1 with one pinnate outer spine and strong spinules; exp-2 with one pinnate inner spine and one outer spine; exp-3 with one pinnate inner spine, one bipinnate seta and spine distally, and two pinnate outer spines. Enp subequal in length to exp; enp-1 and enp-2 each with one spinulose inner spine; enp-3 with two pinnate distal spines, and two serrate outer spines.

Armature formula for swimming legs:

**Table:** 

	Exopod	Endopod
P1	0.1.223	1.1.222
P2	0.1.313	1.1.212
P3	0.1.113	1.1.022
P4	0.1.122	1.1.022

P5 (Figs 1D, 3D) vestigial, represented by four setae; outermost longest and plumose, and innermost smooth.

P6 (Figs 1E, 3B) represented by one long naked seta on each side of genital field.

##### Description of male.

**Figure 9.**
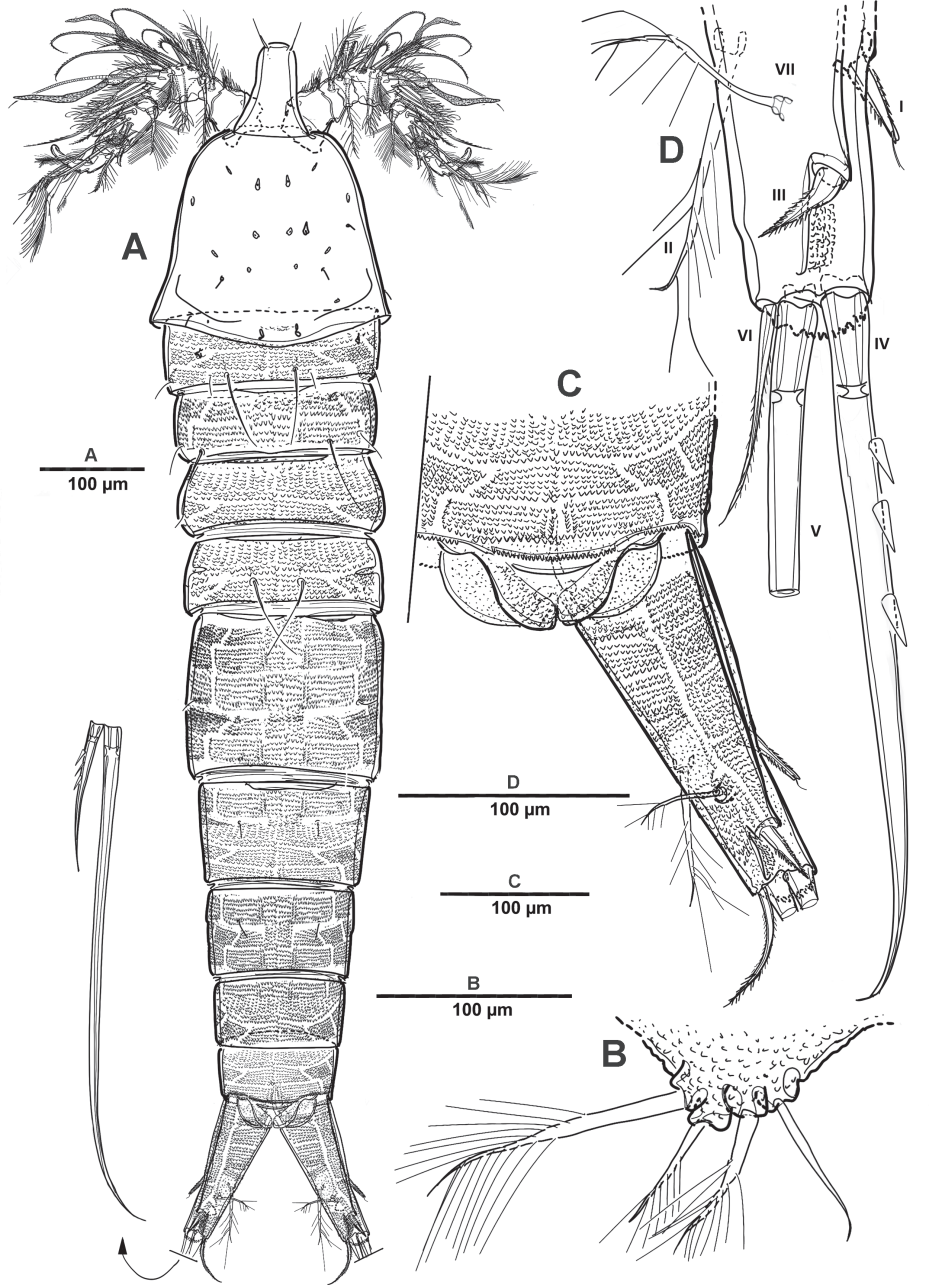
*Elanella jejuensis* sp. nov., paratype male. **A**. Habitus, dorsal; **B**. P5; **C**. Caudal ramus, dorsal; **D**. Caudal ramus, dorsal.

Habitus (Fig. 9A) as in female. Total body length 1,136 µm. Sexual dimorphism mainly in antennule, P2, urosome, and caudal ramus.

**Figure 10.**
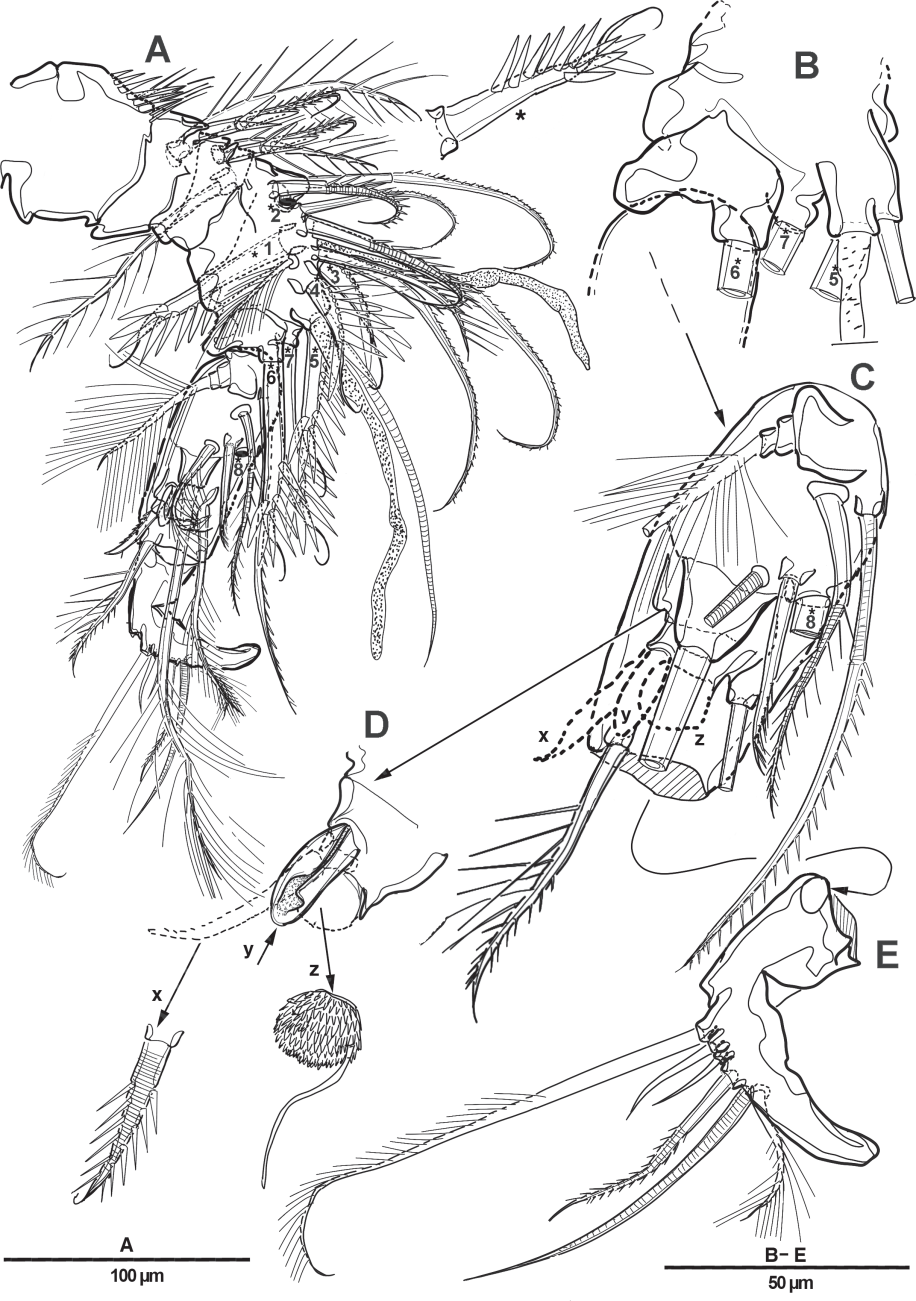
*Elanella jejuensis* sp. nov., paratype male. **A**. Antennule; **B**. Distal part of antennular segment 2; **C**. Antennular segment 3; **D**. Antennular segment 3, detail of distinctive elements (x, y, z); **E**. Antennular segment 4. Asterisks (*) and numerals (1–8) indicate strong pinnate spine-like setae.

Antennule (Fig. 10A–E) chirocer segmentation unclear, presumed 4-segmented. Segment 1 unarmed, with row of spinules and long setules around anterior margin (Fig. 10A). Segment 2 (Fig. 10A, B) with incomplete sutures; with 17 setae/spines and two aesthetascs, including seven strong pinnate spine-like setae (indicated by asterisks and numbered 1–7). Segment 3 (Fig. 10C, D) elliptical and armed with one tri-articulated plumose, three plumose, six pinnate, and one strong pinnate spine-like setae (indicated by an asterisk and numbered 8), as well as three distinctive elements: one strong bipinnate spine-like seta (x), one modified spatulate element (y), and one modified, spinulose element (z) bearing one delicate accessory seta. Segment 4 (Fig. 10E) small, with three pinnate, two bare, and one geniculate setae.

**Figure 11.**
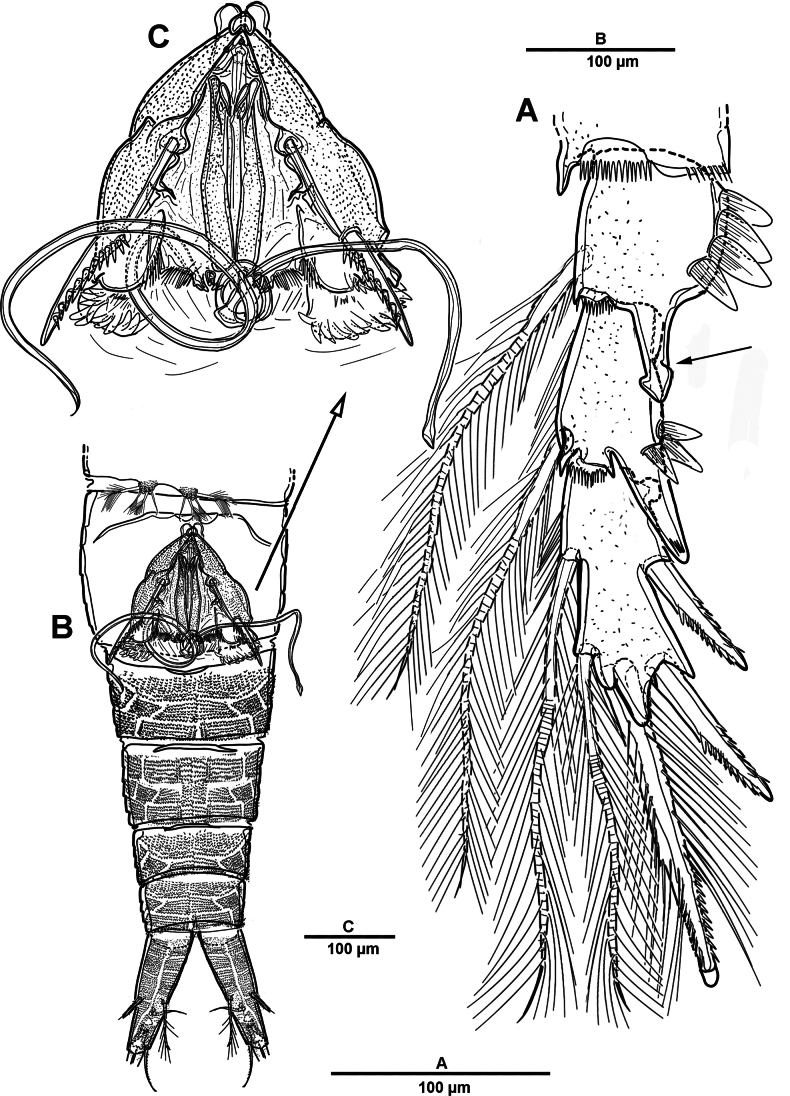
*Elanella jejuensis* sp. nov., paratype male. **A**. P2 enp-1; **B**. Urosome, ventral; **C**. Genital field, ventral.

P2 enp-1 (Fig. 11A) with sagittate process on its outer distal corner (arrowed in Fig. 11A).

Urosome (Fig. 11B) consisting of P5-bearing somite, genital somite, and four postgenital somites. P5 (Fig. 9B) similar to that of female.

Genital somite (Fig. 11B, C) subquadrate, with apertures occluded by fused P6. Each P6 forming one broad plate, with one ventral articulated pinnate element, and one second element from its inner margin. Genital area with supplementary long elements exceeding P6 plates.

Caudal rami (Fig. 9C, D) as in female except for seta IV without bulbiform base.

##### Etymology.

The specific epithet jejuensis refers to the geographic location Jeju-do Island, where the species was discovered.

### Genus *Brianola* Monard, 1926

#### Brianola coreana sp. nov.

Taxon classificationAnimaliaCanuelloidaCanuellidae

E9A9D68E-A523-5459-A877-AF575C0A48DD

https://zoobank.org/033861D5-DD5F-4F45-A460-1F9158E67975

Figs 12, 13, 14, 15, 16, 17, 18, 19, 20, 21

##### Type locality.

Intertidal zone of Gujwa-eup, Jeju-do Island, Korea (33°29'15"N, 126°54'32"E).

##### Type material.

***Holotype*** • 1♀ (MABIK CR00242447) dissected on eight slides. ***Paratypes*** • 1♂ (MABIK CR00242532) dissected on ten slides, • 1 ♀ (MABIK CR00242448) dissected on nine slides, • 5♀♀ (MABIK CR00242533, MABIK CR00242534, MABIK CR00242449 – MABIK CR00242451) preserved in 99% alcohol.

##### GenBank accession numbers.

Mitochondrial cytochrome c oxidase subunit I gene (PP297485, PP297486); 18S ribonucleic acid gene (PP298071, PP298072).

##### Description of female.

**Figure 12.**
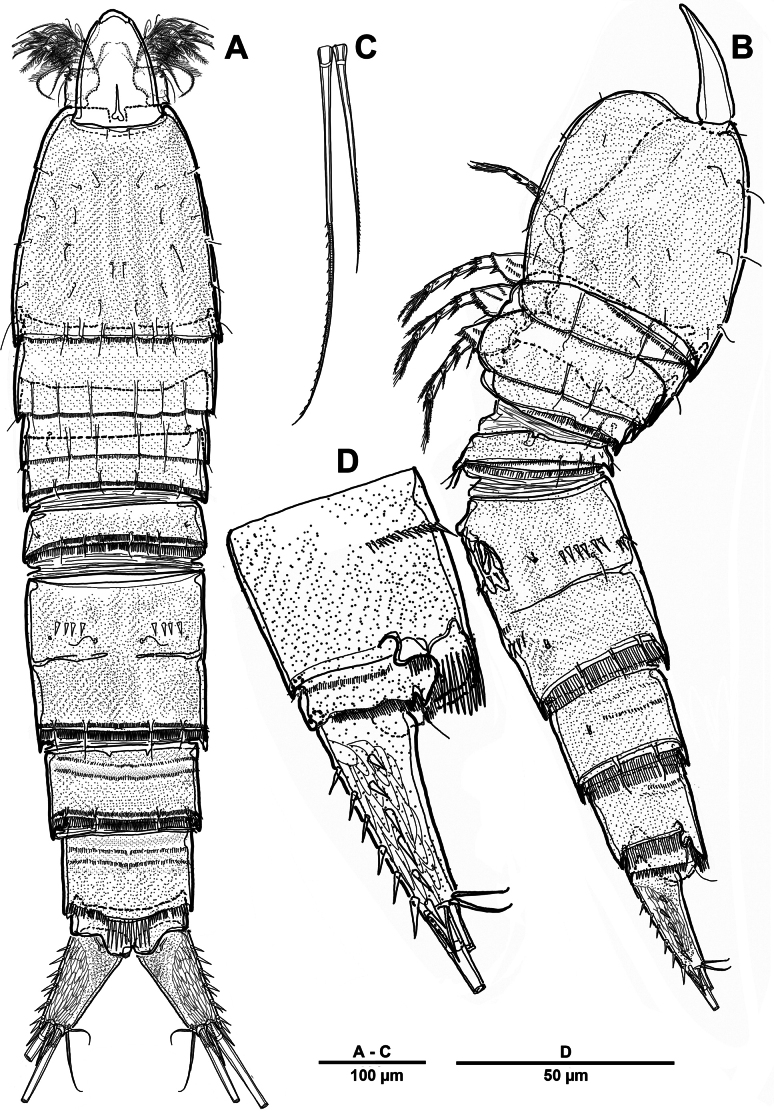
*Brianola coreana* sp. nov., holotype female. **A**. Habitus, dorsal; **B**. Habitus, lateral; **C**. Caudal ramus, dorsal.

Body (Fig. 12A, B) length 968 µm, measured from anterior margin of rostrum to posterior margin of caudal rami. Largest width measured at posterior margin of cephalic shield: 196 µm. Body semi-cylindrical, and entire body covered with minute punctae. First pedigerous somite fused to cephalosome, forming a cephalothorax. Posterior margin of cephalothorax and free prosomites with long setules dorsally and laterally.

**Figure 13.**
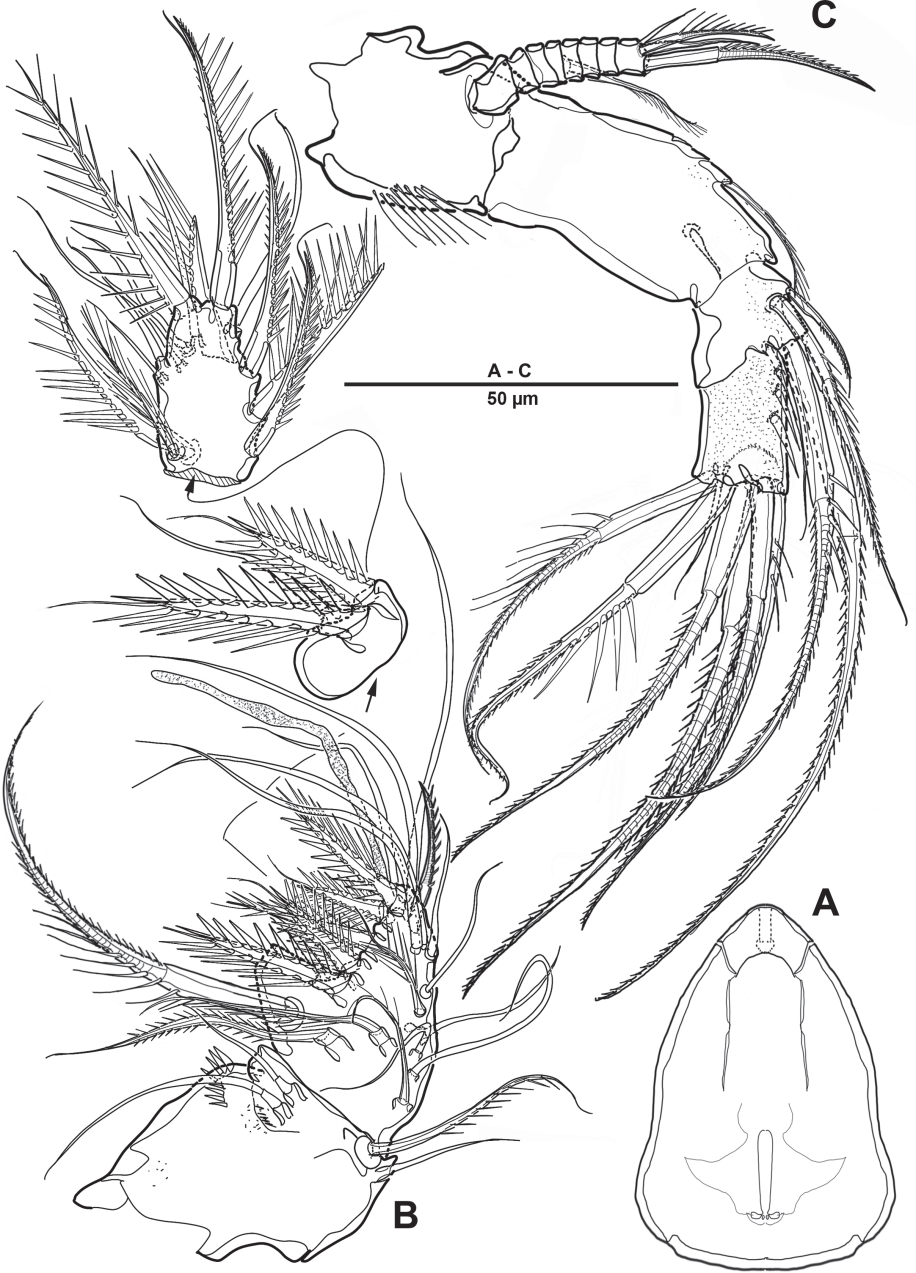
*Brianola coreana* sp. nov., holotype female. **A**. Rostrum; **B**. Antennule; **C**. Antenna (coxa omitted).

Rostrum (Fig. 13A) huge, bell-shaped, defined at base; with subapical pore on each side, tube pore on ventral surface.

**Figure 14.**
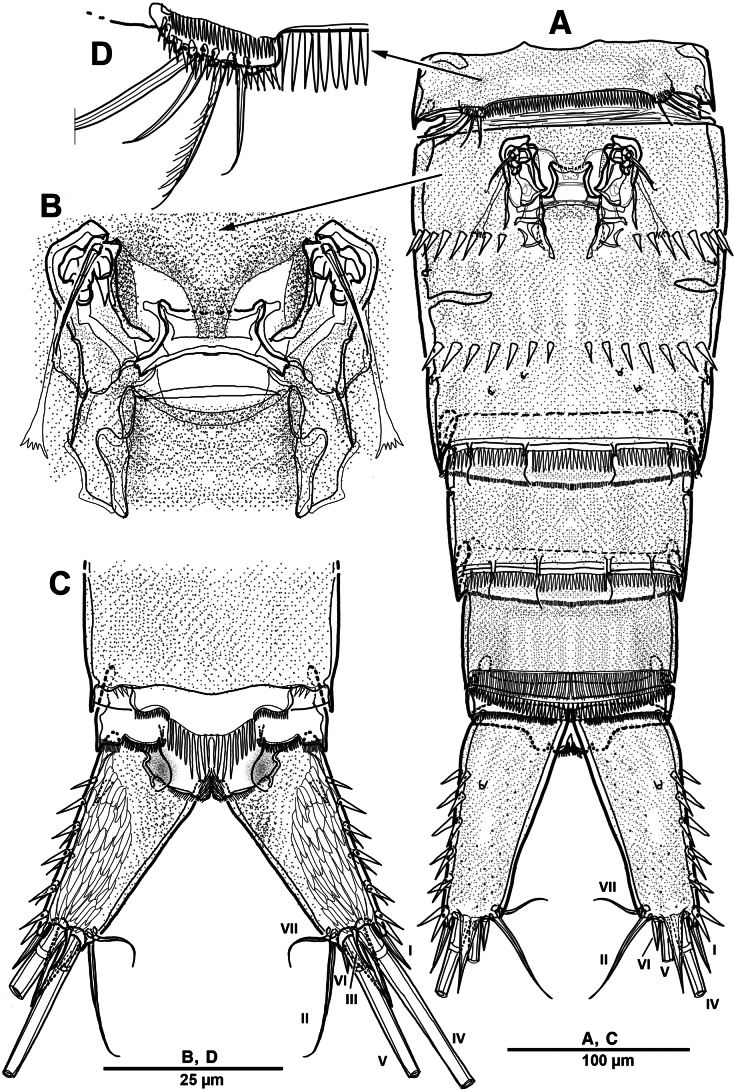
*Brianola coreana* sp. nov., holotype female. **A**. Urosome, ventral; **B**. Genital field and P6; **C**. Anal somite and caudal ramus, dorsal; **D**. P5.

Urosome (Figs 12A, 14A) 5-segmented, comprising P5-bearing somite, genital double-somite and three free urosomites, and dorsal and ventral surfaces covered with small spinules and pores as shown. Genital double-somite marked laterally by continuous internal chitinous band, indicating original division between two somites. Genital ﬁeld (Fig. 14B) located mid-ventrally in anterior half of genital double-somite. P6 represented by paired flaps with one long bare seta on each side of genital field, enclosing gonopores. Anal somite (Fig. 14A, C); pseudoperculum well-developed, arising from penultimate somite, with slightly concave medial margin and fringe of elongate spinules along free margin, not medially cleft.

Caudal rami (Figs 12C, 12D, 14A, 14C) about 2.5 times as long as wide; posteroventral margin drawn out into a spiniform process; dorsal surface with scale-like ornamentation laterally; rows of strong spinules on outer margin and pore on ventral surface. Armature represented by seven setae: seta I spiniform, inserted near outer distal corner; seta II bare and located on distal inner corner; seta III smooth and minute, inserted dorsally between bases of setae IV and V; setae IV and V well-developed and unipinnate; seta V longest; seta VI spiniform, inserted at base of seta V; seta VII bare, and close to seta II.

Antennule (Fig. 13B) 4-segmented. First segment with row of spinules on dorsal surface near outer distal margin, one pinnate and three bare setae. Second segment with eleven naked, eleven pinnate or spinulose elements (three bi-articulate at base), and aesthetasc. Third segment short, with one smooth and two spiniform setae. Distal with seven pinnate, one plumose, and six naked setae. Armature formula: 1-[4], 2-[22 + ae], 3-[3], 4-[14].

Antenna (Fig. 13C) coxa and basis distinct. Basis unornamented, with long setules on abexopodal margin. Exp considerably smaller than enp, 8-segmented; fourth segment with one pinnate seta; distal segment with two bare and two pinnate setae. Enp 3-segmented; enp-1 longest, about 3.1 times as long as enp-2; enp-1 with one pinnate and one smooth setae; enp-2 with two medial and two distal pinnate setae; enp-3 with six pinnate, one minute, and one slender bare setae.

**Figure 15.**
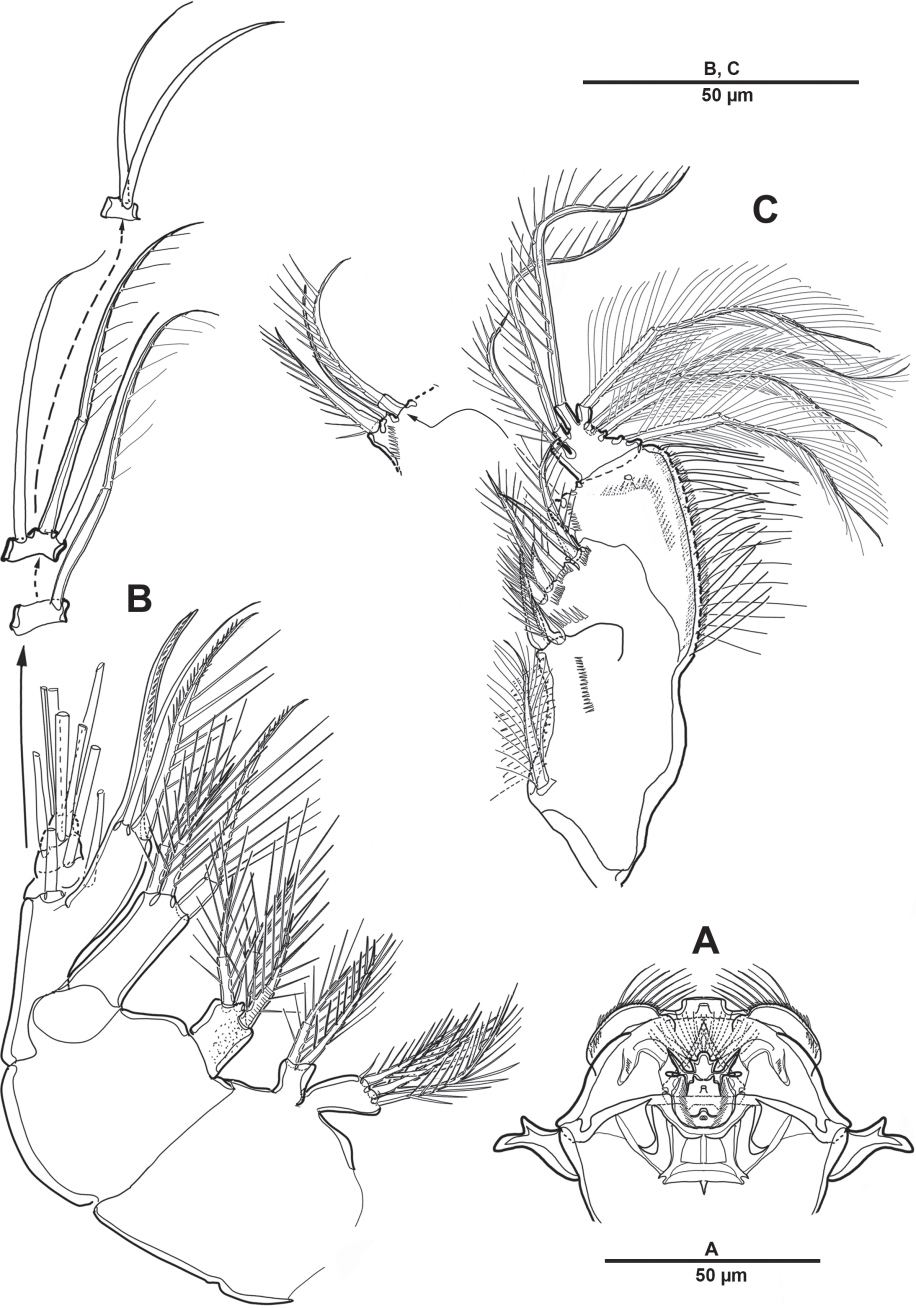
*Brianola coreana* sp. nov., holotype female. **A**. Paragnaths; **B**. Maxilla; **C**. Maxilliped.

Paragnaths (Fig. 15A) developed lobes with one row of long setules.

**Figure 16.**
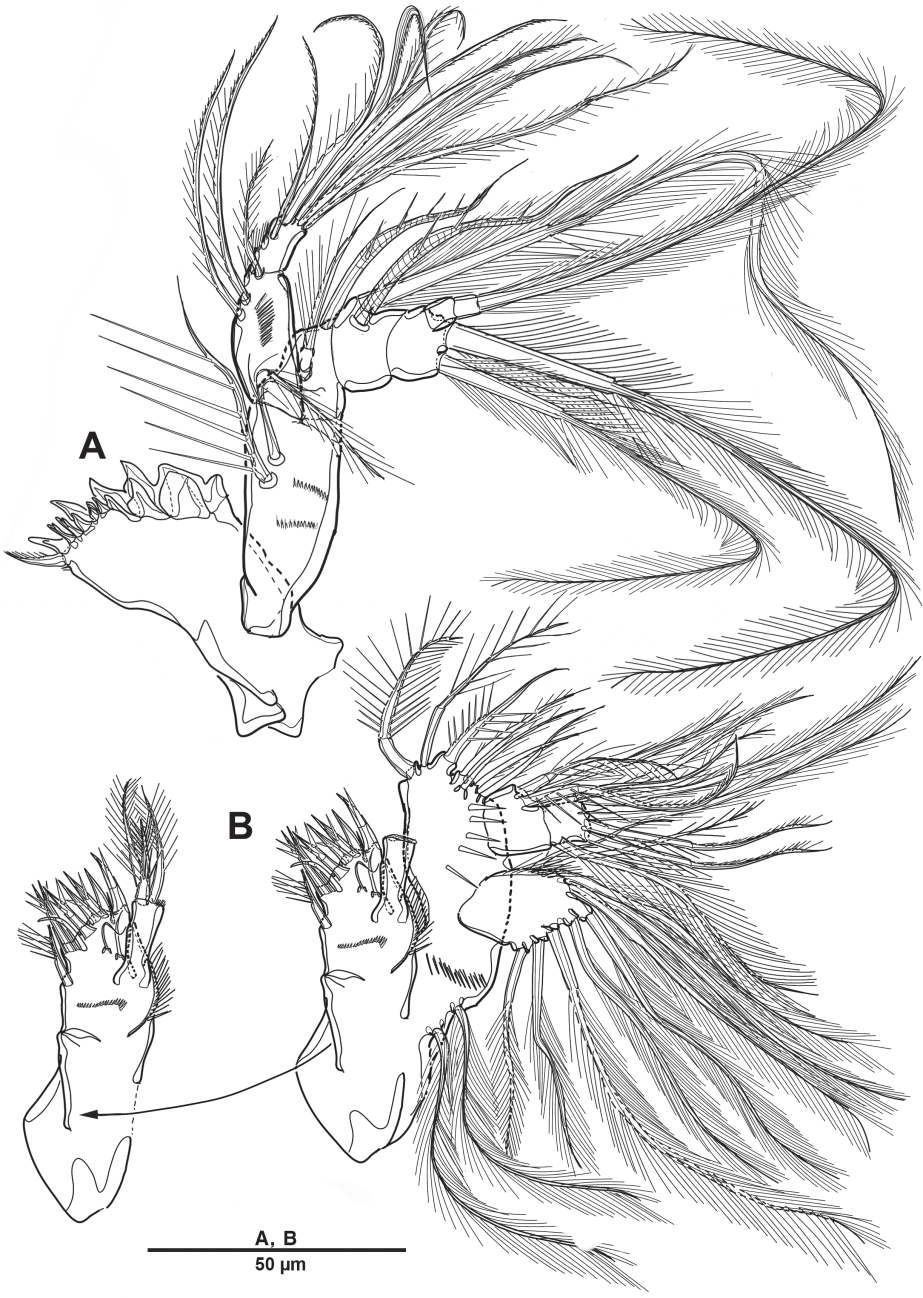
*Brianola coreana* sp. nov., holotype female. **A**. Mandible; **B**. Maxillule.

Mandible (Fig. 16A). Gnathobase with two rows of strong teeth of different size, and dorsal corner with one pinnate seta. Basis with two pinnate setae and two rows of tiny spinules. Exp 3-segmented; exp-1 and exp-2 each with one plumose and one pinnate setae; exp-3 with one proximal tri-articulate and two distal plumose setae. Enp 2-segmented; enp-1 with three pinnate setae and row of spinules; enp-2 with six pinnate and two plumose setae.

Maxillule (Fig. 16B). Praecoxa and coxa partially fused. Praecoxal arthrite with eight spines and two setae around distal margin, and two setae on anterior surface. Coxal endite cylindrical, with three pinnate setae; with two long plumose epipodal setae. Basis with closely set endites, proximal endite with three pinnate setae, distal endite with four pinnate setae. Exp unsegmented; with eight plumose and two pinnate setae. Enp 2-segmented; enp-1 with two plumose and two pinnate setae laterally; enp-2 with four plumose and two pinnate setae.

Maxilla (Fig. 15B) comprising praecoxa, coxa, allobasis and 3-segmented enp. Praecoxa bearing two endites; proximal endite with three pinnate setae; distal endite with two pinnate setae. Coxa with two endites of which proximal somewhat shorter, each with three pinnate setae. Allobasis drawn out into one strong pinnate claw, with one spinulose spine and one bare seta. Enp 3-segmented; enp-1 with one unipinnate seta; enp-2 with one pinnate and two naked setae; enp-3 with two bare setae.

Maxilliped (Fig. 15C) phyllopodial, 2-segmented, with undivided protopod and 1-segmented enp; incomplete suture marks boundary between syncoxa and basis. Protopod with one praecoxal plumose seta, seven coxal setae/spines, and three basal setae; with rows of small spinules as illustrated; outer margin with long setules. Enp with seven setae; three inner setae and apical seta pinnate, four outer setae plumose.

**Figure 17.**
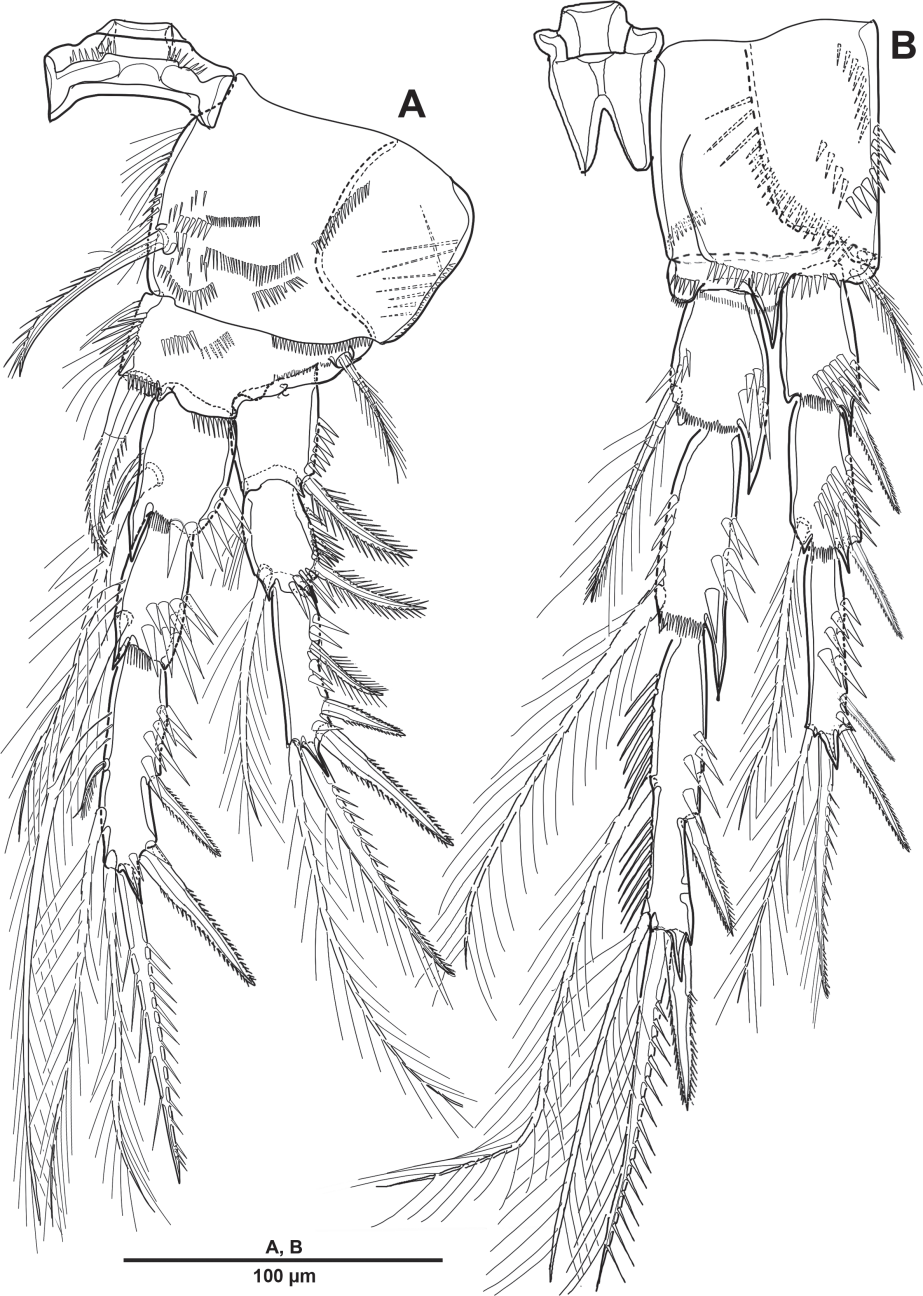
*Brianola coreana* sp. nov., holotype female. **A**. P1; **B**. P2.

**Figure 18.**
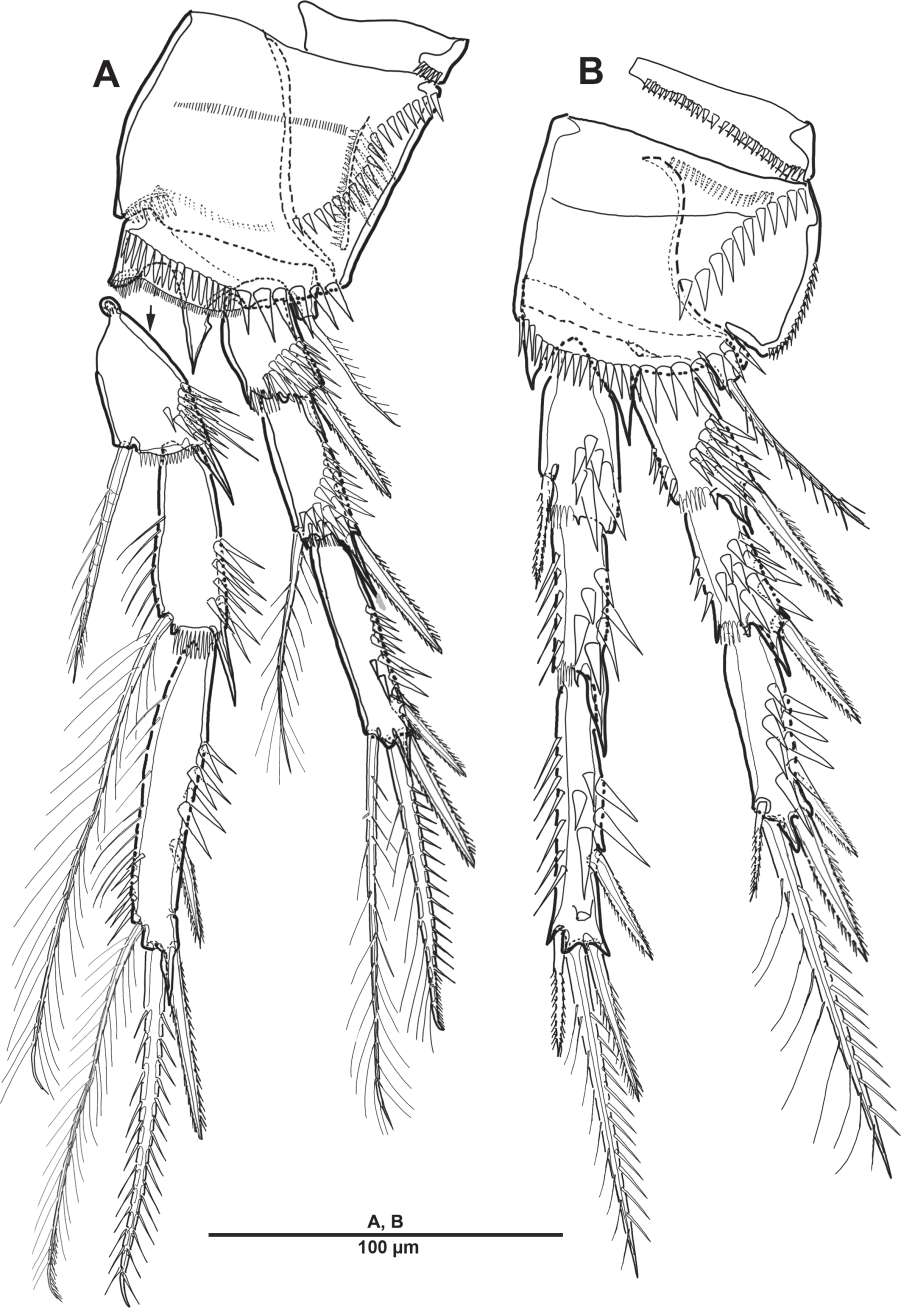
*Brianola coreana* sp. nov., holotype female. **A**. P3; **B**. P4.

Swimming legs 1–4 (Figs 17, 18) biramous, both rami 3-segmented, with intercoxal sclerite, unarmed praecoxae, coxae, and bases. Coxa with spinular rows on anterior surface as figured. Enp always longer than exp.

P1 (Fig. 17A). Intercoxal sclerite transversely elongate. Coxa with long setules along inner margin, rows of spinules on anterior surface, and one inner long pinnate seta. Basis with rows of spinules and setules as shown, one bipinnate outer seta, and one strong bipinnate inner spine. Exp-1–3 with spinular ornamentation as shown; exp-1 with one outer bipinnate spine; exp-2 with one plumose inner seta and one outer pinnate spine; exp-3 with one plumose seta and one bipinnate spine apically, and three pinnate outer elements. All endopodal segments with long setules along inner margin, and strong spinules on anterior surface; enp-1 and enp-2 each with one long plumose inner seta; enp-3 with one small medial and one long plumose inner setae, two distal elements (one plumose seta and one stout bipinnate spine), and two serrate outer spines.

P2 (Fig. 17B). Intercoxal sclerite well-developed and unornamented. Coxa unarmed with rows of spinules as figured. Basis with one bipinnate outer seta, posterior surface with spinous processes between two rami. Exp-1 and exp-2 each with two rows of spinules along outer distal margin, and outer distal corner forming spinous process; exp-1 with one pinnate outer spine; exp-2 with one inner plumose seta and one outer spine; exp-3 with two distal pinnate setae, and two pinnate outer elements. Enp-1 and enp-2 each with one inner plumose seta, patch of outer spinules, and outer distal corner forming spinous process; enp-3 with two plumose inner setae, two apical elements, one serrate outer spine, and rows of spinules on outer and inner margin.

P3 (Fig. 18A) similar to P2 except for setal formulae of enp-3. Enp-3 with two distal pinnate elements, and two serrate outer spines.

P4 (Fig. 18B). Coxa rectangular, with rows of spinules on surface. Basis similar to P2 and P3. Exp-1 and exp-2 each with one pinnate outer spine and strong spinular patch at their base; exp-3 with strong spinules on anterior surface, one small inner pinnate and one strong terminal bipinnate setae, and two pinnate outer spines. Enp-1 with small pinnate inner seta, and strong spinules on anterior surface; enp-2 with outer apophysis developed; enp-3 with one inner spiniform seta, two distal elements (one distal inner spiniform seta and one distal outer spine), one outer spine, and anterior surface with pore.

Armature formula for swimming legs:

**Table:** 

	Exopod	Endopod
P1	0.1.023	1.1.222
P2	0.1.022	1.1.221
P3	0.1.022	1.1.122
P4	0.0.112	1.0.121

P5 (Fig. 14D) vestigial, incorporated into somite, represented by four setae, outermost seta bare and longest, and second innermost seta pinnate.

##### Description of male.

**Figure 19.**
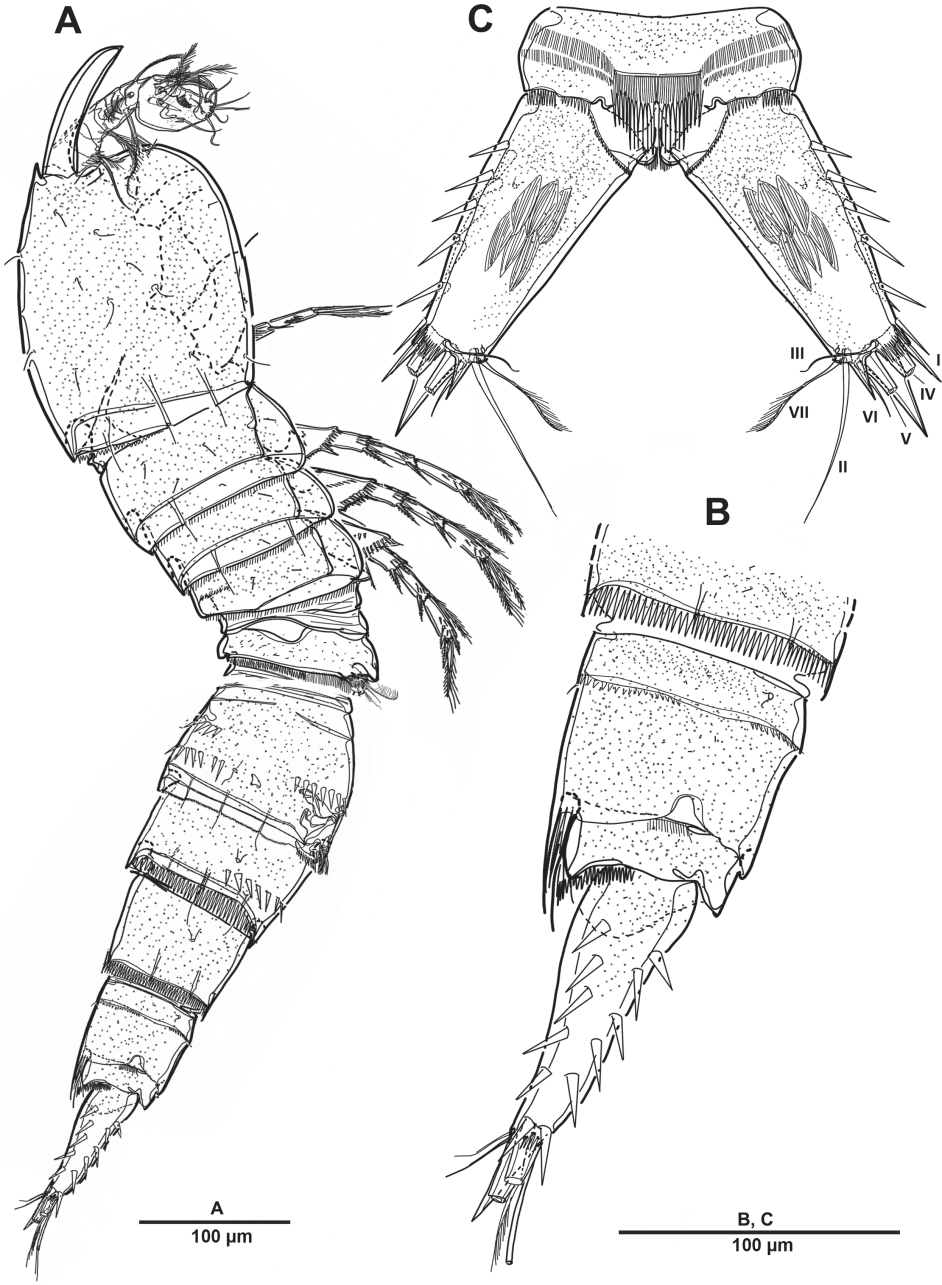
*Brianola coreana* sp. nov., paratype male. **A**. Habitus, dorsal; **B**. Caudal ramus, lateral; **C**. Caudal rami, dorsal.

Habitus (Fig. 19A). Total body length 795 µm. Sexual dimorphism mainly in antennule, P5, urosome and caudal ramus.

**Figure 20.**
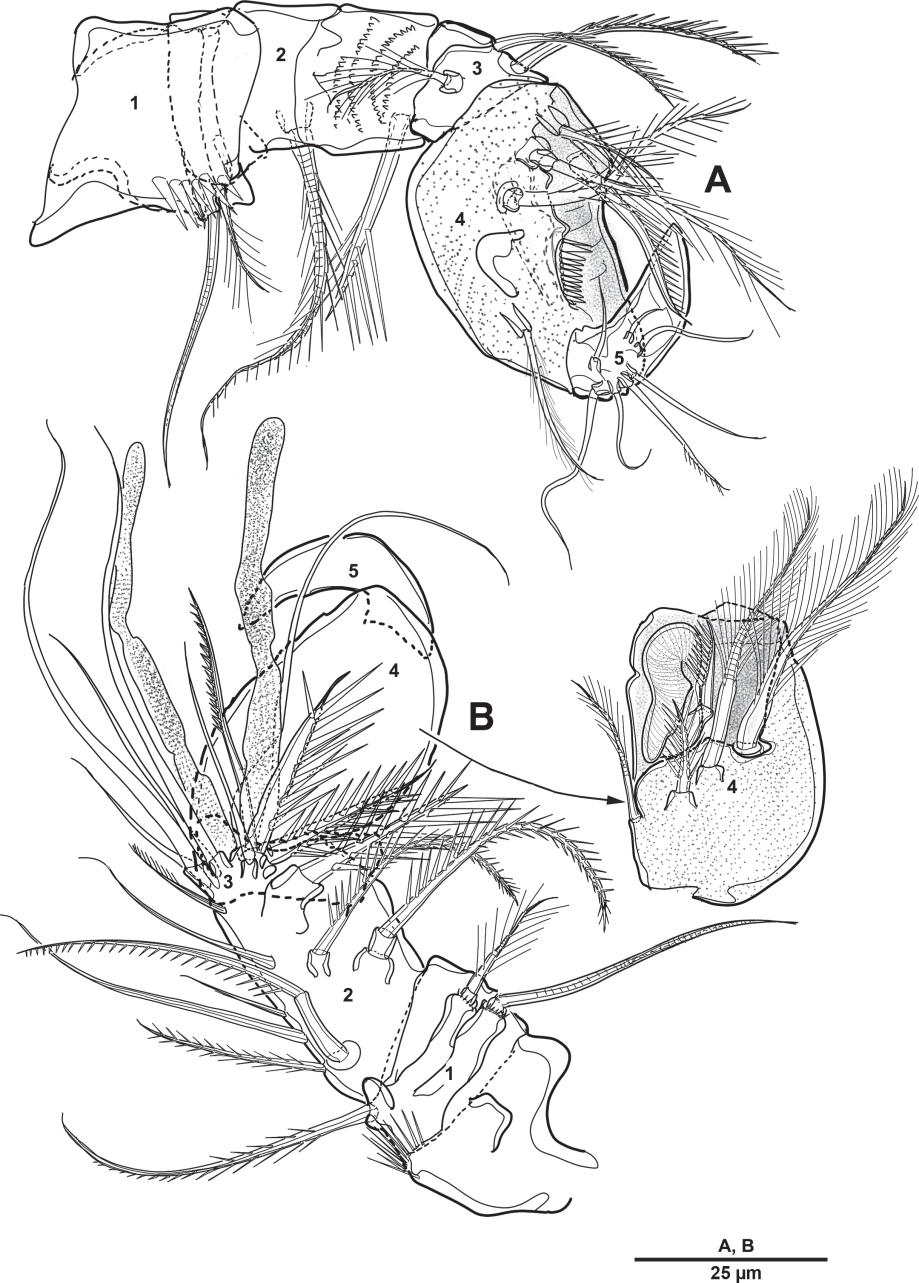
*Brianola coreana* sp. nov., paratype male. **A**. Antennule, dorsal; **B**. Antennule, ventral. Arabic numerals (1–5) indicate segments 1–5.

Antennule (Fig. 20A, B) 5-segmented, chirocer; geniculation between segments 4 and 5. Segments 1–5 indicated by Arabic numerals (Fig. 20A, B). Segment 1 with two bipinnate and one smooth setae, with two rows of spinules. Segment 2 with transverse sutures but no functional articulations, with ten pinnate and nine smooth setae and two aesthetascs. Segment 3 small, with one plumose and two pinnate setae. Segment 4 swollen and largest, with five plumose and five pinnate setae, and one corrugated pad ventrally; dorsal surface with row of stout spinules. Segment 5 with one pinnate and six bare setae, apex conical, slightly incurved, blunt.

**Figure 21.**
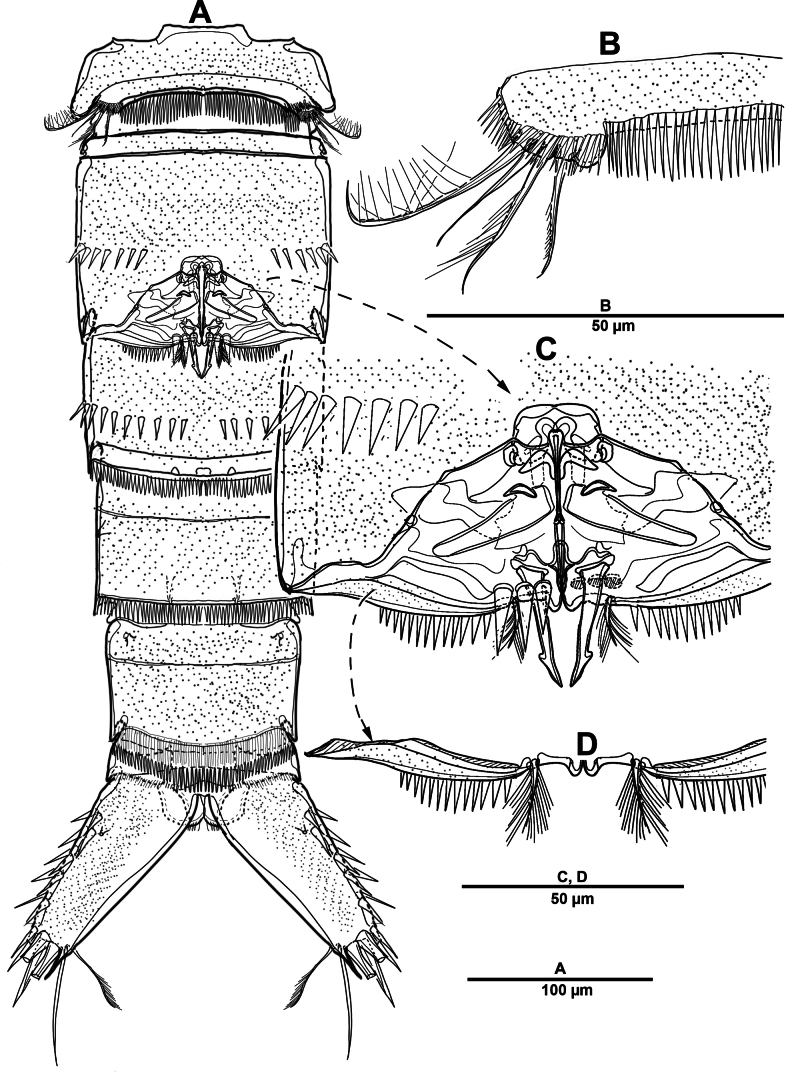
*Brianola coreana* sp. nov., paratype male. **A**. Urosome and P5, ventral; **B**. P5 (detail); **C**. Genital field; **D**. P6.

P5 (Fig. 21A, B) with armature as in female, but innermost and outermost setae plumose.

Urosome (Fig. 21A) slightly tapering posteriorly; consisting of P5-bearing somite, genital somite, three free urosomites, and anal somite; with spinules and pores as illustrated.

Genital somite quadrate, without lateral spinous extension. Genital field (Fig. 21C) with rows of stout spinules on posterior margin; P6 (Fig. 21D) represented by one small bipinnate seta on each side of genital field, accompanied by three strong spinules and an arrowhead-like element.

Caudal rami (Fig. 19B, C) as in female except for seta VII plumose.

##### Etymology.

The species epithet indicates the collection locality, South Korea.

### Genus *Scottolana* Huys, 2009

#### Scottolana picrca sp. nov.

Taxon classificationAnimaliaCanuelloidaCanuellidae

BEA5E4AE-04E8-55E5-ACAF-C9BBF1171DF1

https://zoobank.org/757B3835-A53A-427F-9EB5-D1CC611D88E9

Figs 22, 23, 24, 25, 26, 27, 28, 29, 30, 31, 32

##### Type locality.

Subtidal zone near Riptide beach, Malkal, Koror, Republic of Palau (7°19'48"N, 134°27'20"E).

##### Type material.

***Holotype*** • 1♀ (MABIK CR00259509) dissected on twelve slides. ***Paratypes*** •1♂ (MABIK CR00259512) dissected on eight slides, • 1 ♀ (MABIK CR00259508) dissected on fourteen slides. • 2♀♀ (MABIK CR00259510, MABIK CR00259511) preserved in 99% alcohol.

##### GenBank accession numbers.

Mitochondrial cytochrome c oxidase subunit I gene (PP297489 – PP297491); 18S ribonucleic acid gene (PP298075 – PP298077).

##### Description of female.

**Figure 22.**
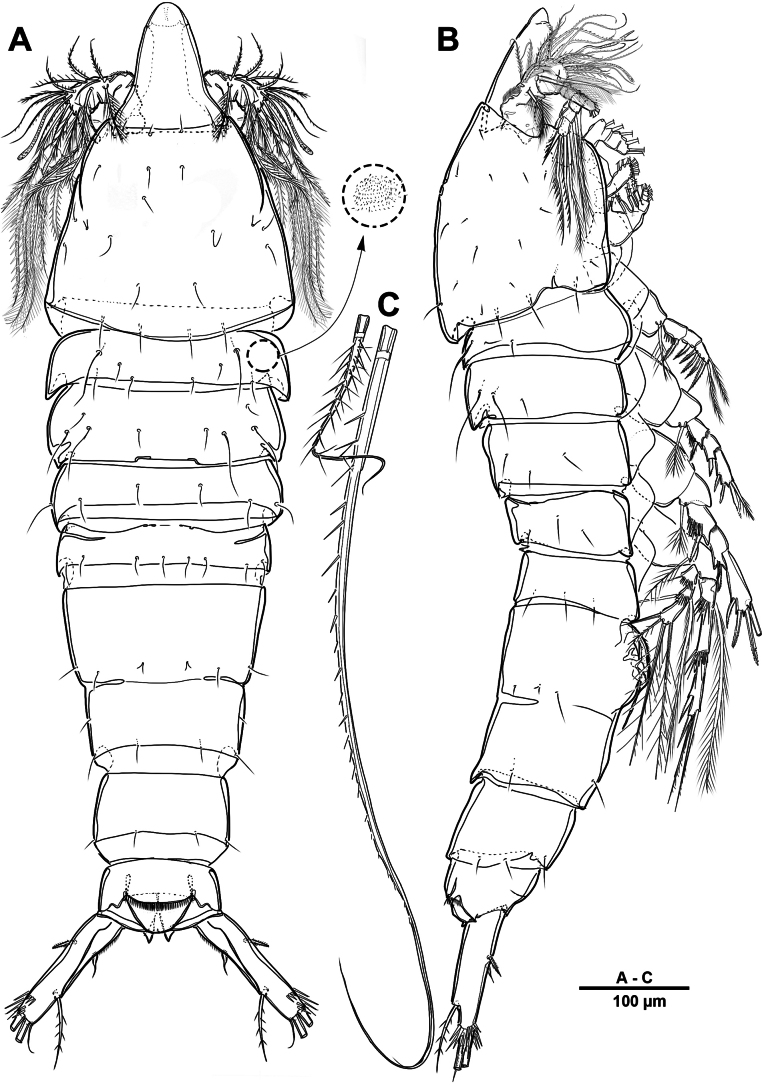
*Scottolana picrca* sp. nov., holotype female. **A**. Habitus, dorsal; **B**. Habitus, lateral, **C**. Caudal setae IV and V.

**Figure 23.**
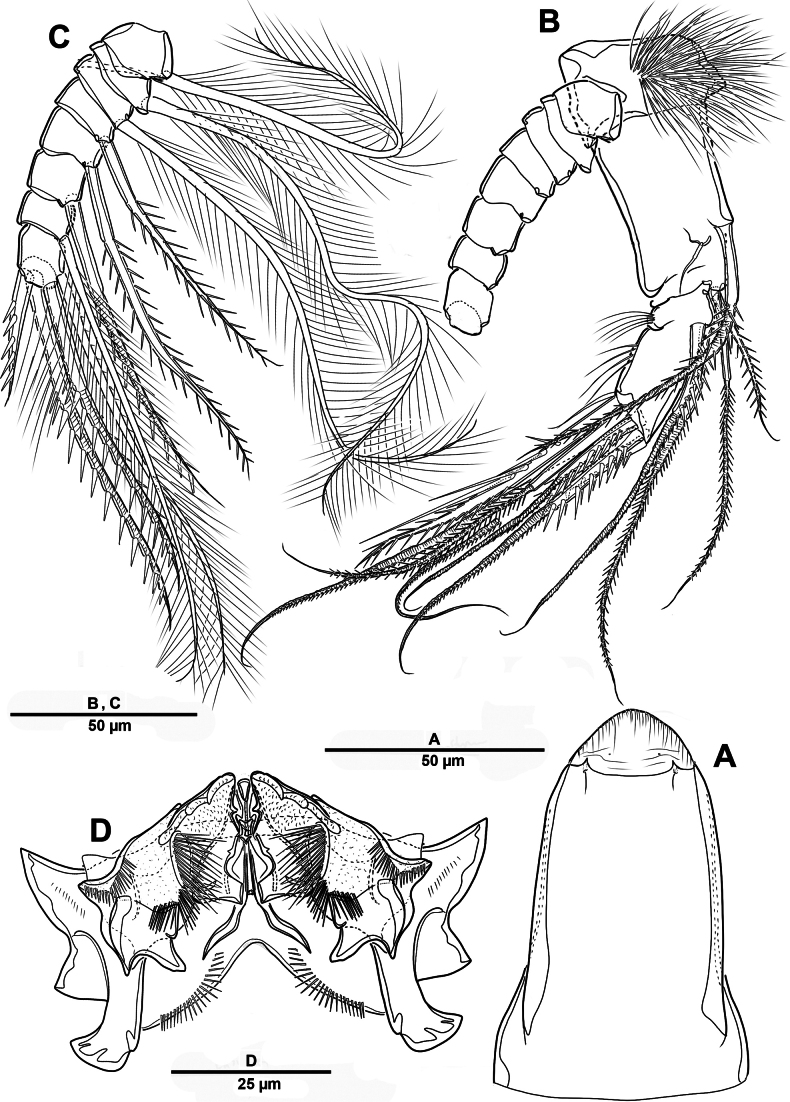
*Scottolana picrca* sp. nov., holotype female. **A**. Rostrum; **B**. Antenna; **C**. Antennal exopod; **D**. Paragnaths.

Body (Fig. 22) length 908 µm, measured from anterior margin of rostrum to posterior margin of caudal rami. Largest width measured at posterior margin of cephalic shield: 224 µm. Body semi-cylindrical, entire surface densely covered with minute spinules as figured, comprising 5-segmented prosome and 5-segmented urosome. Cephalosome and free prosomites with long sensilla on surface. First free prosomite separated from cephalosome. Rostrum (Figs 22A, 23A) prominent, more than half length of cephalic shield; with two sensillae, with blunt apex.

**Figure 24.**
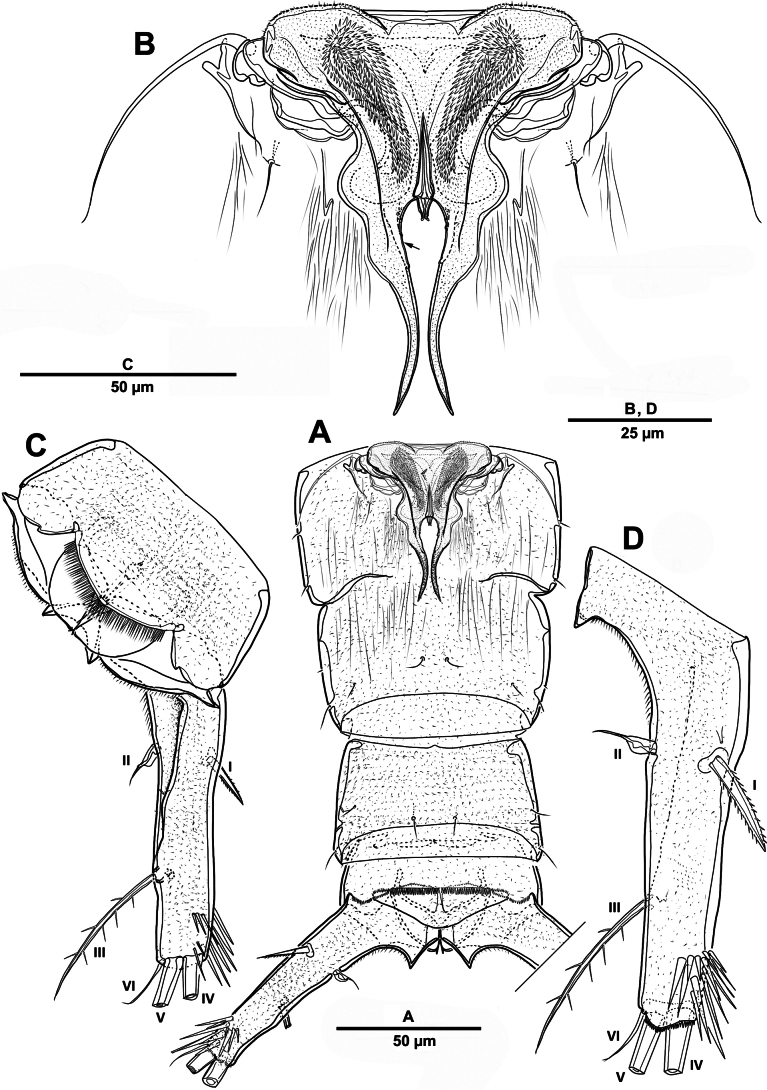
*Scottolana picrca* sp. nov., holotype female. **A**. Urosome, ventral; **B**. Genital field and P6; **C**. Anal somite and caudal ramus, dorsal; **D**. Caudal ramus, ventral.

Urosome (Figs 22A, 22B, 24A) consisting of P5-bearing somite, genital double-somite and two free urosomites, slightly narrower than prosome, with ventral surface covered with small spinules. Genital ﬁeld (Fig. 24B) enlarged, extending to about mid-length of genital double-somite. Copulatory pores paired (arrowed in Fig. 24B), opening on inner margin of scythe-like processes. Gonopores located near anterior edge of genital field, each closed off by P6 operculum bearing one long bare seta. Proximal part of genital plate ornamented with paired denticulate patches.

Anal somite (Fig. 24A, C) with lateral process on each side, anal operculum well-developed, semicircular, with fine setules along distal margin. Caudal rami (Figs 22C, 24C, 24D) measure ~ 2.5 times as long as wide. Each ramus bears seven setae: seta I spiniform and pinnate, located ventrally; seta II short, bare, and inserted on proximal third with bulbiform base; seta III bipinnate and positioned dorsally on distal third of ramus; seta IV well-developed, tripinnate (Fig. 22C); seta V unipinnate, longest; seta VI short and bare; seta VII pinnate, bi-articulate at base, inserted near seta III as in male (not illustrated; damaged).

**Figure 25.**
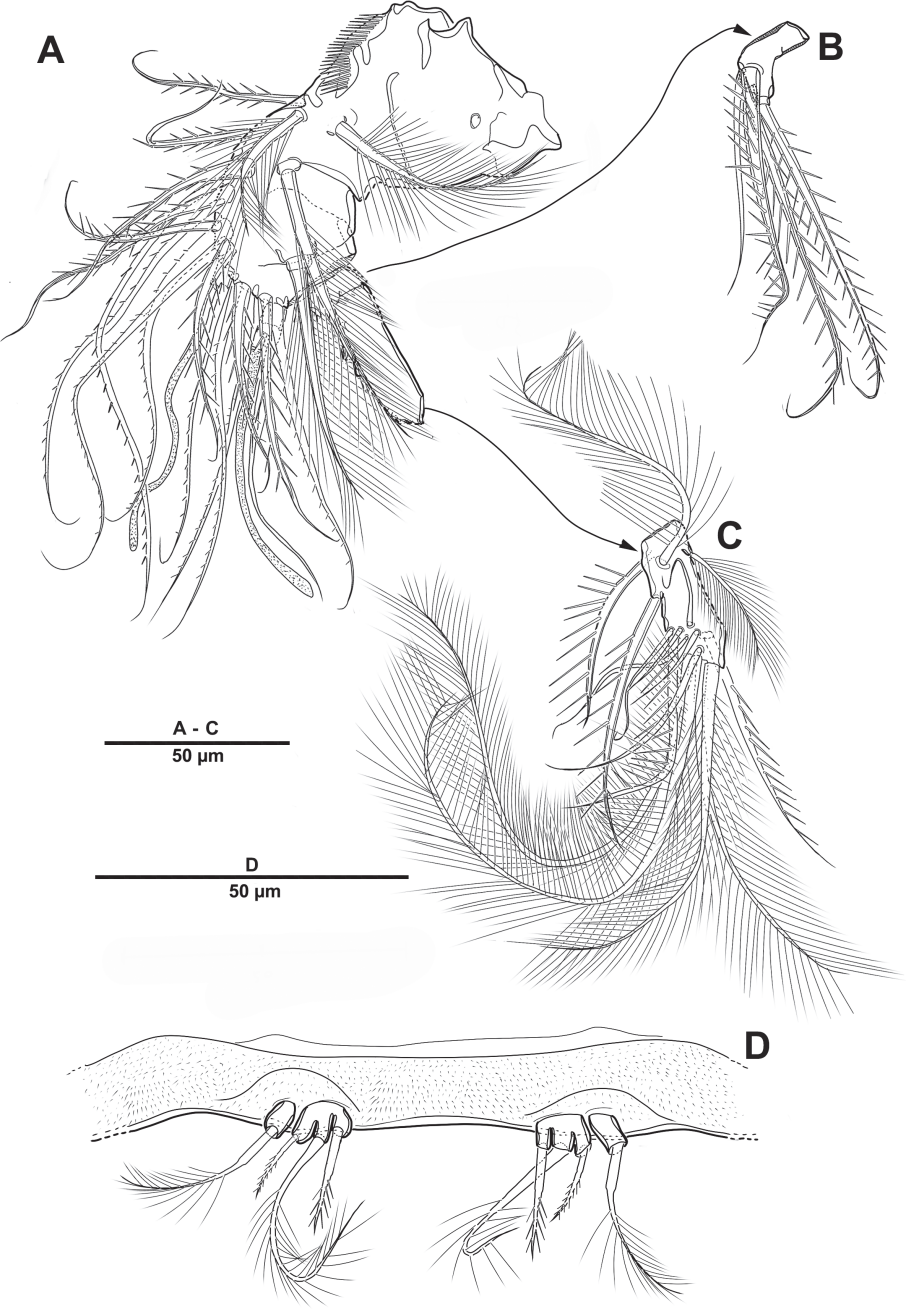
*Scottolana picrca* sp. nov., holotype female. **A**. Antennule; **B**. Antennular segment 2; **C**. Antennular segments 3; **D**. P5.

Antennule (Fig. 25A–C) 3-segmented. First segment largest, with three incomplete transverse sutures around posterior margin; with 21 setae/spines and two aesthetascs. Middle segment short, with one naked and three pinnate setae. Distal segment with four long plumose and two pinnate setae apically, three plumose/pinnate and six naked setae on anterior margin, and one plumose seta posteriorly. Armature formula: 1-[21 + 2ae], 2-[4], 3-[16].

Antenna (Fig. 23B, C) comprising short coxo-basis, 2-segmented enp, and 8-segmented exp. Coxa and basis fused to form short coxo-basis, with long setules on anterior surface. Enp 2-segmented; enp-1 with one strong pinnate and one small smooth setae; enp-2 with incomplete transverse ridge indicating original segmentation, with four lateral pinnate and seven distal setae (one of them small and bare). Exp 8-segmented; segments 1–3 and 7 each with one plumose seta, and segments 4–6 each with one bipinnate seta; distal segment with three pinnate and one plumose setae.

Paragnaths (Fig. 23D) well-developed, trilobed, with complex pattern of long setules as figured.

**Figure 26.**
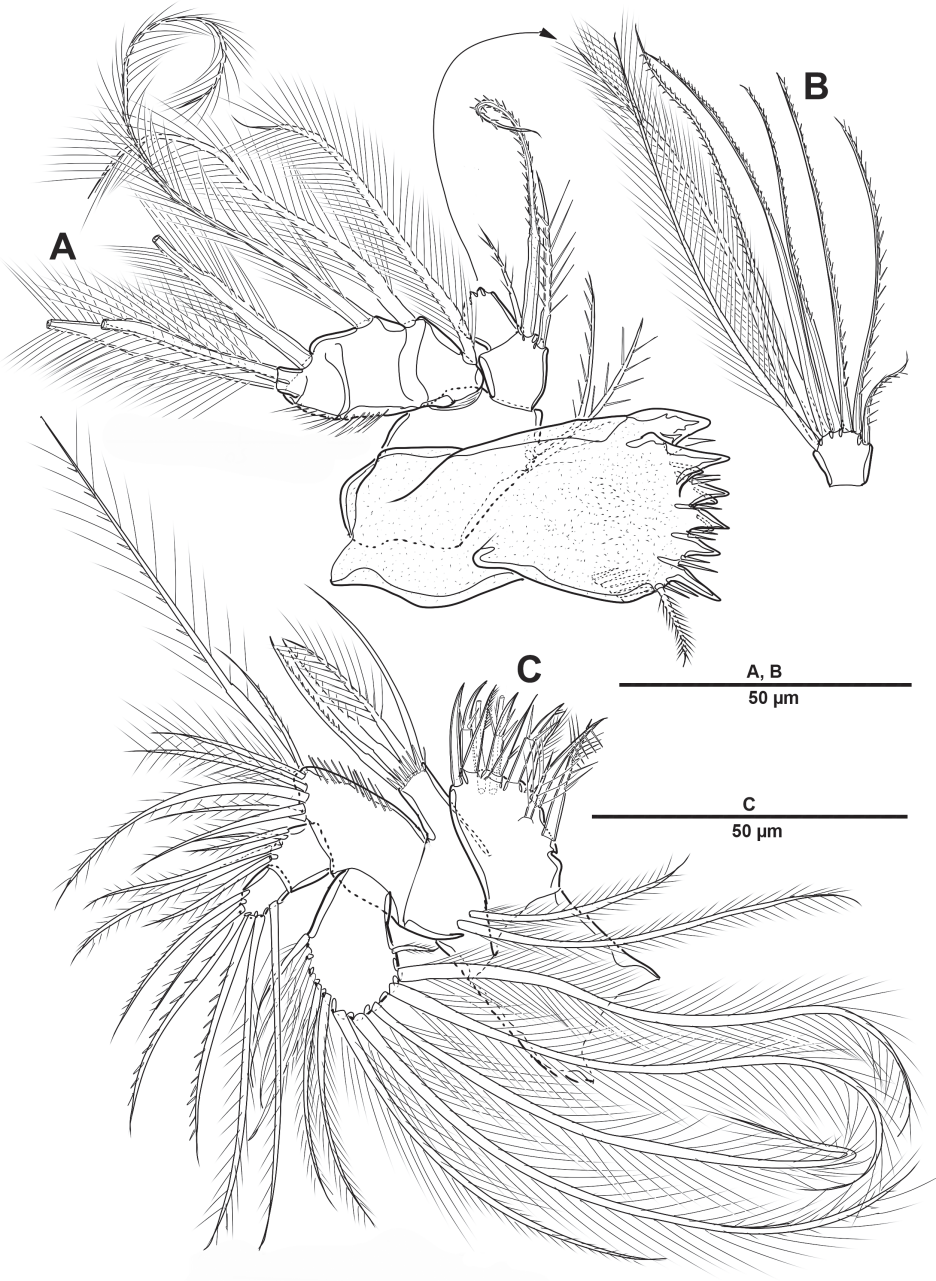
*Scottolana picrca* sp. nov., holotype female. **A**. Mandible; **B**. Mandibular enp-2; **C**. Maxillule.

Mandible (Fig. 26A, B). Gnathobase with two rows of strong teeth of different size, and with one pinnate seta on dorsal corner. Basis with two bipinnate setae. Exp 3-segmented, all setae long and plumose; exp-1 with two setae; exp-2 and exp-3 each with row of spinules at outer margin; exp-3 with one proximal and two distal setae. Enp 2-segmented; enp-1 with three pinnate setae; enp-2 with two plumose and six pinnate setae (Fig. 26B).

Maxillule (Fig. 26C). Praecoxa partly fused to coxa. Praecoxal arthrite with eight spines and two setae on distal margin, and two juxtaposed setae on anterior surface. Coxal epipodite represented by two plumose setae; coxal endite cylindrical, with two pinnate, one plumose, and one smooth setae. Basis with seven pinnate setae, and one row of setules on posterior surface. Exp foliaceous, with six setae on inner margin, five long setae on distal margin, and one small seta on outer margin, all setae plumose. Enp 2-segmented; enp-1 with five pinnate setae laterally; enp-2 with six pinnate setae.

**Figure 27.**
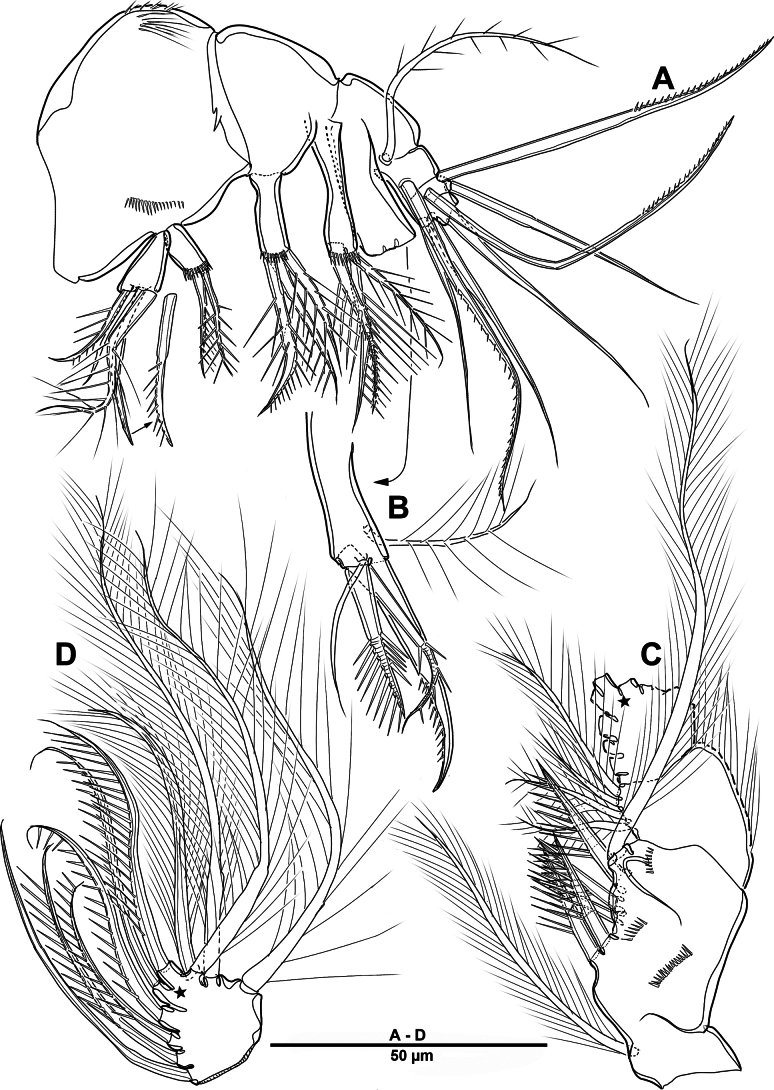
*Scottolana picrca* sp. nov., holotype female. **A**. Maxilla; **B**. Maxillary allobasis elements (detail); **C**. Maxilliped; **D**. Maxillipedal endopod.

Maxilla (Fig. 27A, B). Praecoxa with row of spinules on anterior surface; proximal endite with three pinnate and one smooth setae; distal endite with two pinnate setae. Coxa with two cylindrical endites, each with three pinnate setae. Allobasis drawn out into one strong unipinnate claw, with two strong spinulose spines, one bare seta, and one plumose seta (Fig. 27B). Enp 3-segmented; enp-1 with one plumose and two spiniform setae; enp-2 with one spinulose and two naked setae; enp-3 with two bare setae.

Maxilliped (Fig. 27C, D) phyllopodial, 3-segmented, comprising syncoxa, basis and unsegmented enp. Syncoxa with rows of small spinules on surface, one plumose seta proximally, five pinnate spines on inner margin, and one long plumose seta and one small bare seta near inner distal corner. Basis with four plumose setae. Enp with one naked, five pinnate, and four plumose setae (Fig. 27D).

**Figure 28.**
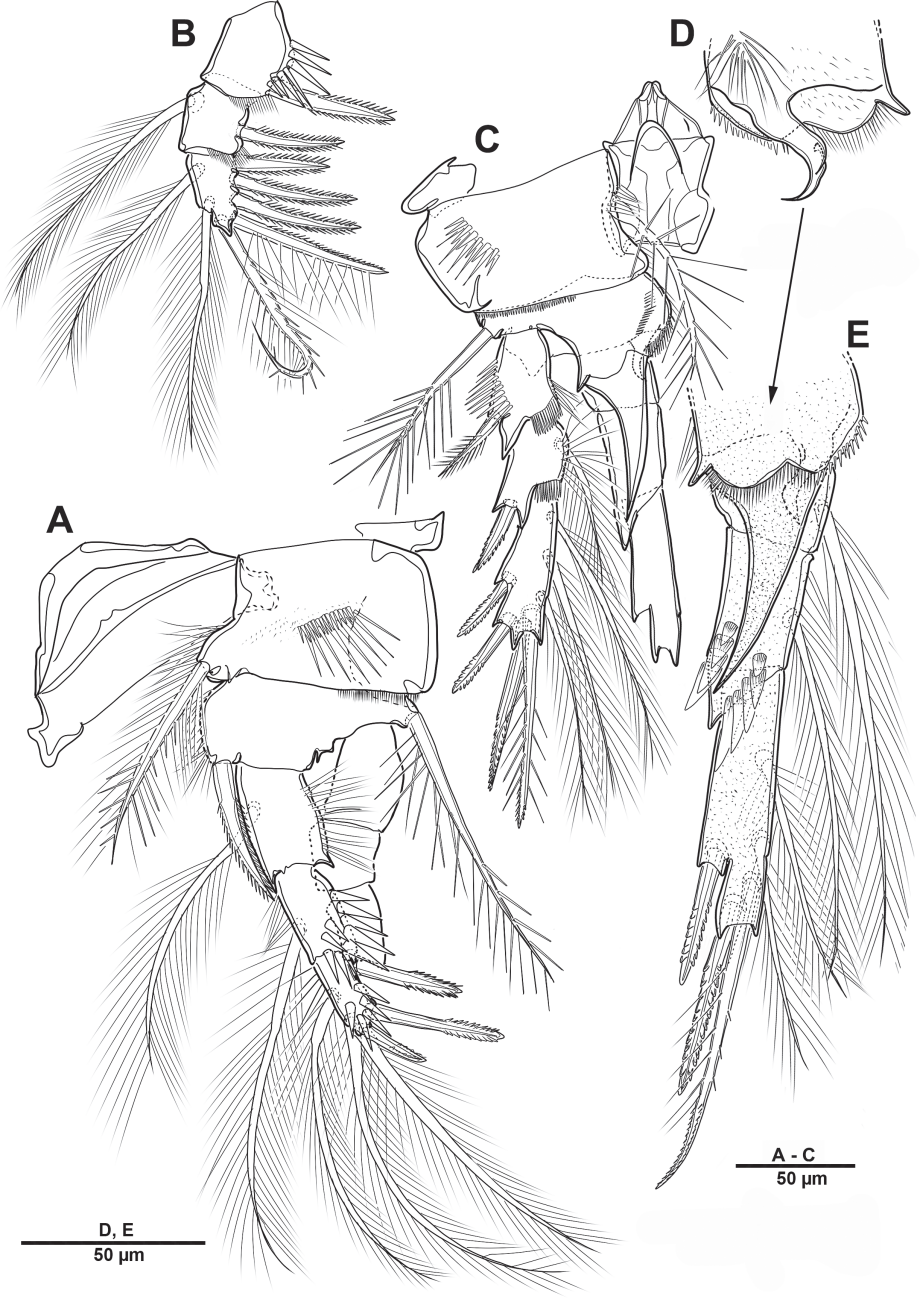
*Scottolana picrca* sp. nov., holotype female. **A**. P1; **B**. P1 exopod; **C**. P2; **D**. P2 basis, posterior; **E**. P2 endopod (detail).

**Figure 29.**
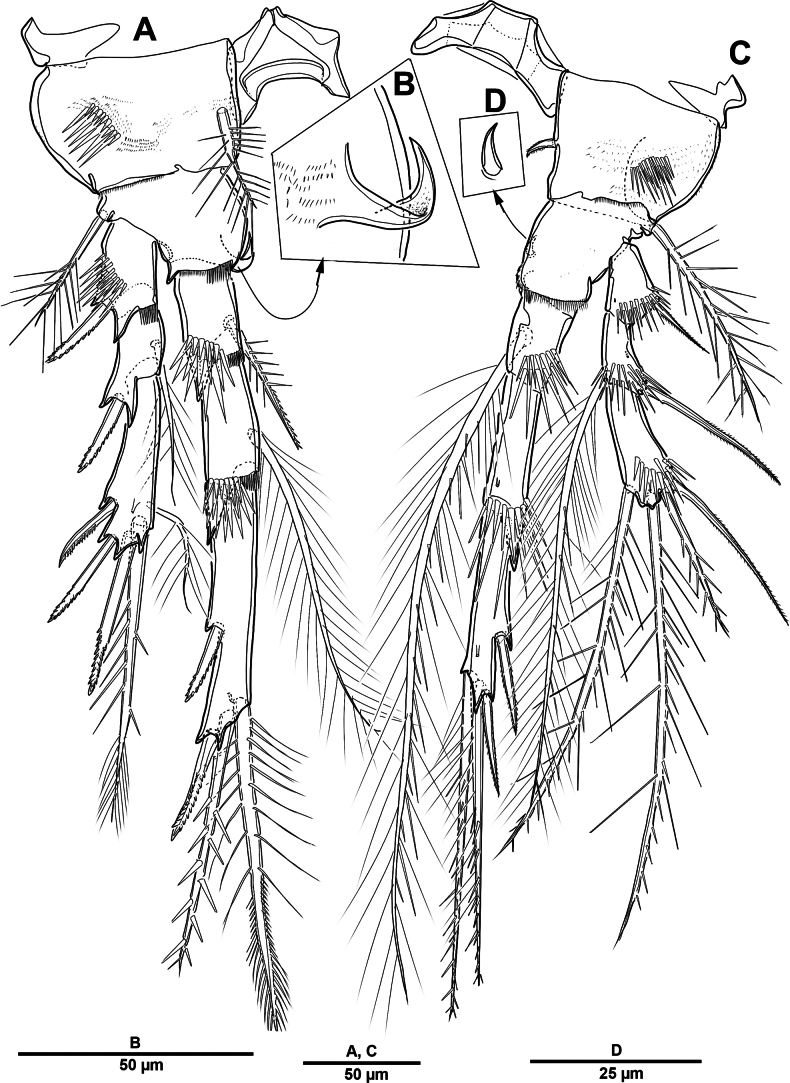
*Scottolana picrca* sp. nov., holotype female. **A**. P3; **B**. P3 basal process, posterior; **C**. P4; **D**. P4 basal process, posterior.

Swimming legs 1–4 (Figs 28, 29) biramous, both rami 3-segmented, with intercoxal sclerite, small unarmed praecoxae, rectangular coxae, and bases. Coxa with spinular rows on anterior surface.

P1 (Fig. 28A, B). Coxa with rows of spinules on anterior surface, long setules at inner distal corner, and one pinnate inner seta. Basis with long setules along inner margin, one pinnate long outer seta, and one bipinnate inner spine. Exp (Fig. 28B) slightly shorter than enp; exp-1 with one outer bipinnate spine, and with spinules around outer margin; exp-2 with one plumose inner seta and one outer pinnate spine; exp-3 with four pinnate spines and three plumose setae. Enp-1 with long setules along outer margin, and one long plumose inner seta; enp-2 with one long inner plumose seta, and with strong spinules on outer distal corner; enp-3 with three long plumose inner setae, one small distal spine, and two serrate outer spines, and with strong spinules on distal and outer margins.

P2 (Fig. 28C–E). Coxa large, with one inner pinnate seta, and row of setules near base of that seta. Basis with one pinnate outer seta, and curved acute process at inner distal corner posteriorly (Fig. 28D). Exp-1 with one pinnate outer spine, row of spinules along outer margin, and setules on inner distal margin; exp-2 with one plumose inner seta and one pinnate outer spine; exp-3 with three plumose inner setae, two distal spines, and two serrate spines. Enp-1 small, with one plumose inner seta, anterior surface produced into spinous process, reaching to distal margin of enp-2; enp-2 with one plumose inner seta, and two rows of strong spinules on anterior surface; enp-3 with two plumose inner setae, two apical spines, and one serrate outer spine.

P3 (Fig. 29A, B). Coxa with one pinnate inner seta, and rows of spinules on anterior surface. Basis with one pinnate outer seta, and setules near inner distal corner; posterior surface with curved processes (Fig. 29B). Exp-1 with one serrate outer spine, and spinules on outer margin; exp-2 with one short plumose inner seta and one serrate outer spine; exp-3 with one plumose inner seta, two apical spines, and two outer spines. Enp much longer than exp, enp-1 and enp-2 each with spinular patch near outer distal corner; enp-1 with one bipinnate spine; enp-2 with one long plumose seta; enp-3 with two distal strong pinnate and two outer serrate spines.

P4 (Fig. 29C, D). Coxa with spinular patch on anterior surface, and one small inner spine. Basis elongate, with one pinnate outer seta, and small spinous process on posterior surface (Fig. 29D). Exp-1 and exp-2 with spinular patch near distal edge; exp-1 with one pinnate outer spine; exp-2 with one plumose inner seta, and one pinnate outer spine; exp-3 with one long bipinnate inner seta, two pinnate apical setae, and pinnate outer spine. Enp much longer than exp; enp-1 with one very long plumose inner seta; enp-2 unarmed, spinular patch and pore on anterior surface; enp-3 with two long spinulose setae apically, and two outer spines.

Armature formula for swimming legs:

**Table:** 

	Exopod	Endopod
P1	0.1.223	1.1.312
P2	0.1.322	1.1.221
P3	0.1.122	1.1.022
P4	0.1.121	1.0.022

P5 (Fig. 25D) vestigial, incorporated into somite, represented by four bipinnate setae, outermost seta separated from remaining setae, and second innermost longest.

##### Description of male.

**Figure 30.**
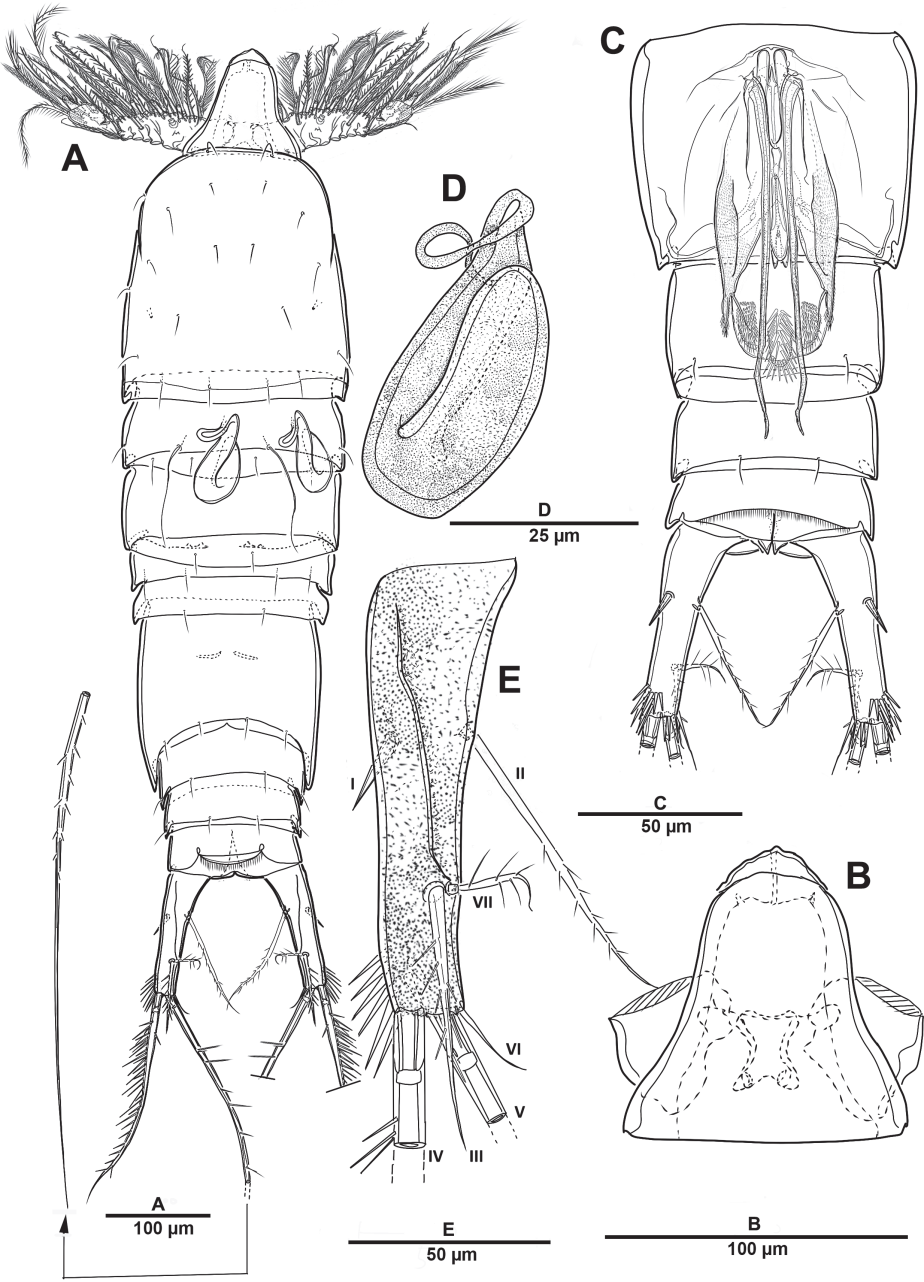
*Scottolana picrca* sp. nov., paratype male. **A**. Habitus, dorsal; **B**. Rostrum; **C**. Urosome, ventral; **D**. Spermatophore; **E**. Caudal ramus, dorsal.

Habitus (Fig. 30A). Total body length 902 µm. Sexual dimorphism mainly in antennule, P3, P5, urosome and caudal ramus.

Rostrum (Fig. 30B) folded, slightly shorter than that of female.

**Figure 31.**
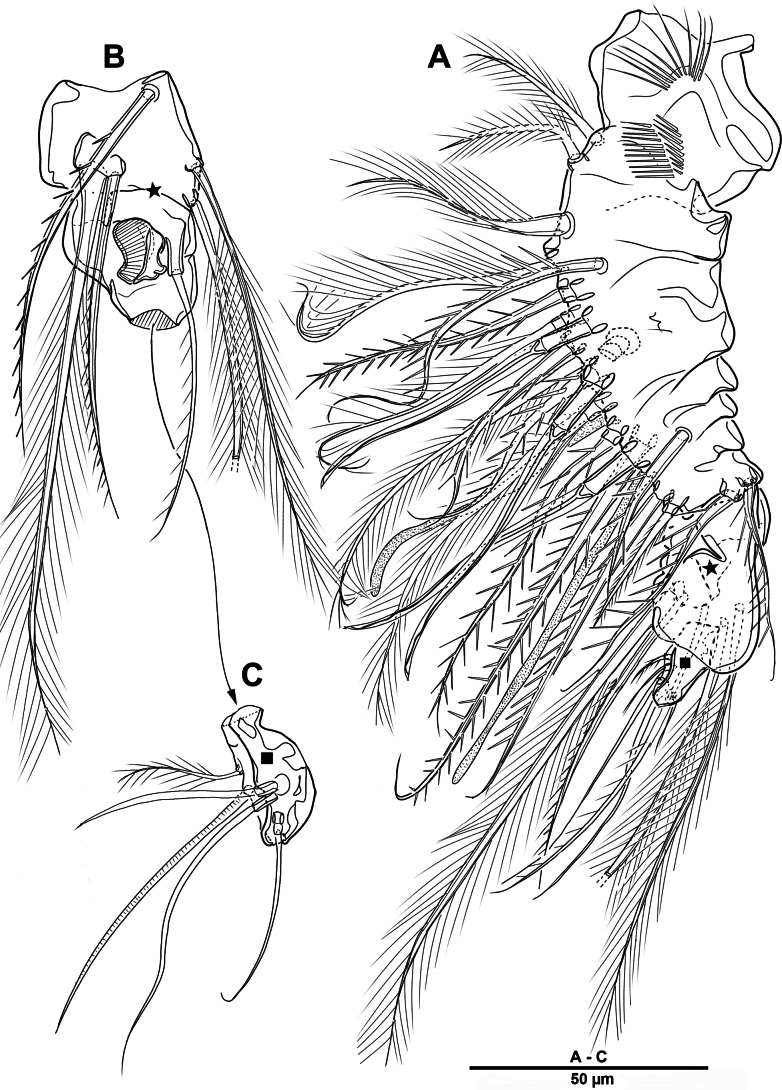
*Scottolana picrca* sp. nov., paratype male. **A**. Antennule; **B**. Antennular segment 2; **C**. Antennular segment 3.

Antennule (Fig. 31A) 3-segmented. Segment 1 very large, with sutures around posterior margin, three rows of spinules, and 22 setae/spines (one tri-articulate, and four bi-articulate at base) and two aesthetascs. Segment 2 (Fig. 31B) with an incomplete suture around posterior margin, with three pinnate and three plumose setae, and one modified ribbed element. Segment 3 (Fig. 31C) small, with one small plumose, one geniculate, two bare, and one tri-articulated smooth setae.

**Figure 32.**
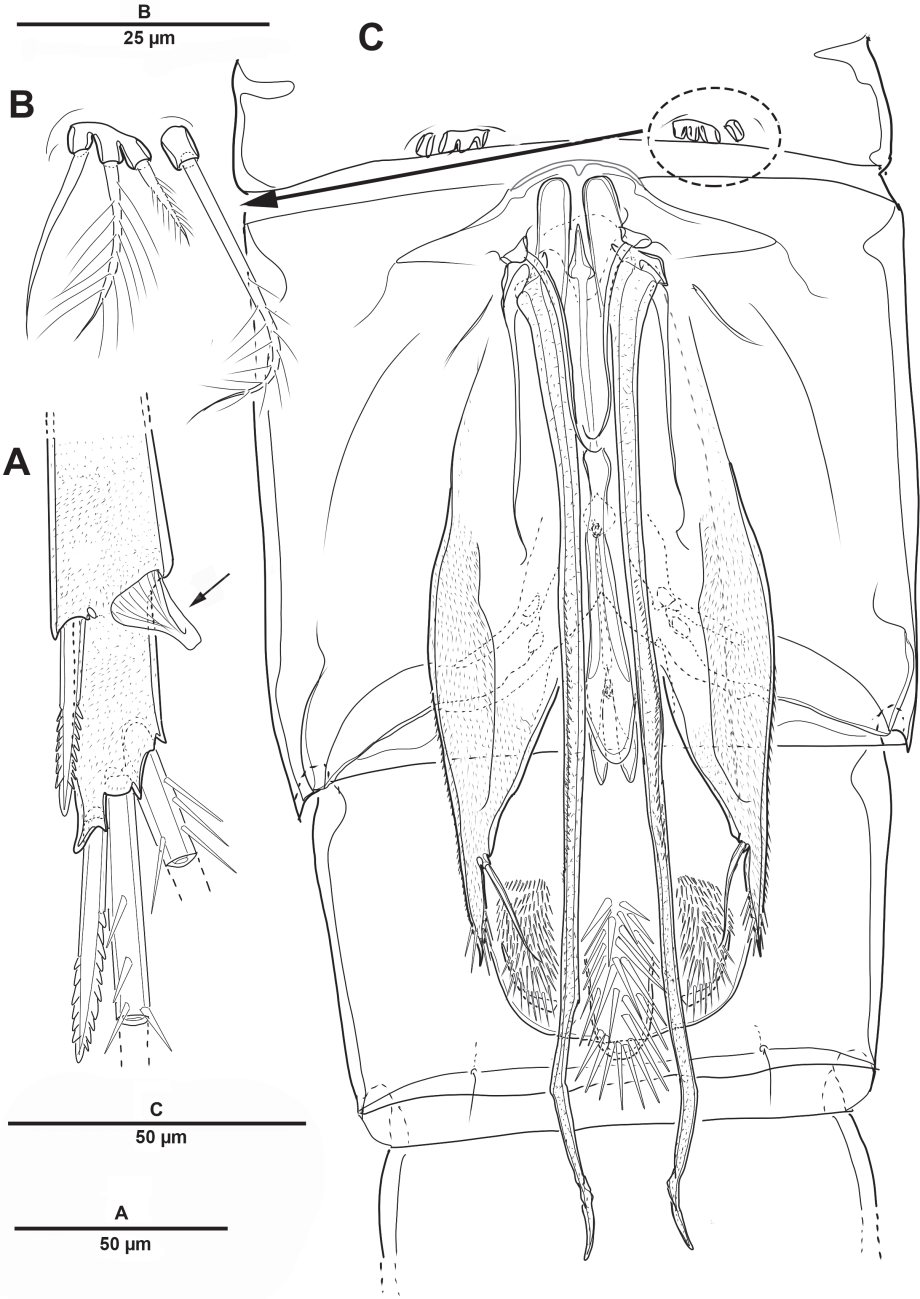
*Scottolana picrca* sp. nov., paratype male. **A**. P3 enp-3; **B**. P5; **C**. Genital somite and first free urosomite, ventral.

P3 enp-3 (Fig. 32A) with large modiﬁed pore at distal half of segment (arrowed in Fig. 32A).

P5 (Fig. 32B) with armature as in female, but innermost seta smooth and outermost seta longest.

Urosome (Fig. 30C) 5-segmented, consisting of P5-bearing somite, genital somite, and three free urosomites. Genital somite (Figs 30C, 32C) about as long as wide, without spinous extension; genital field with extremely long barbed element emerging near proximal margin of P6; P6 forming a large process bearing one bare subapical inner seta, and closing off each genital aperture. First free urosomite with spinulose ridge on ventral surface (Fig. 32C).

Spermatophore (Fig. 30D) paired, with narrow and curved neck.

Caudal rami (Fig. 30E) as in female except for seta I smooth, and seta II not modified, long and pinnate.

##### Etymology.

The species name is derived from the abbreviation of the ‘Palau International Coral Reef Center’, a research institute in the Republic of Palau.

### Phylogenetic analysis

**Figure 33.**
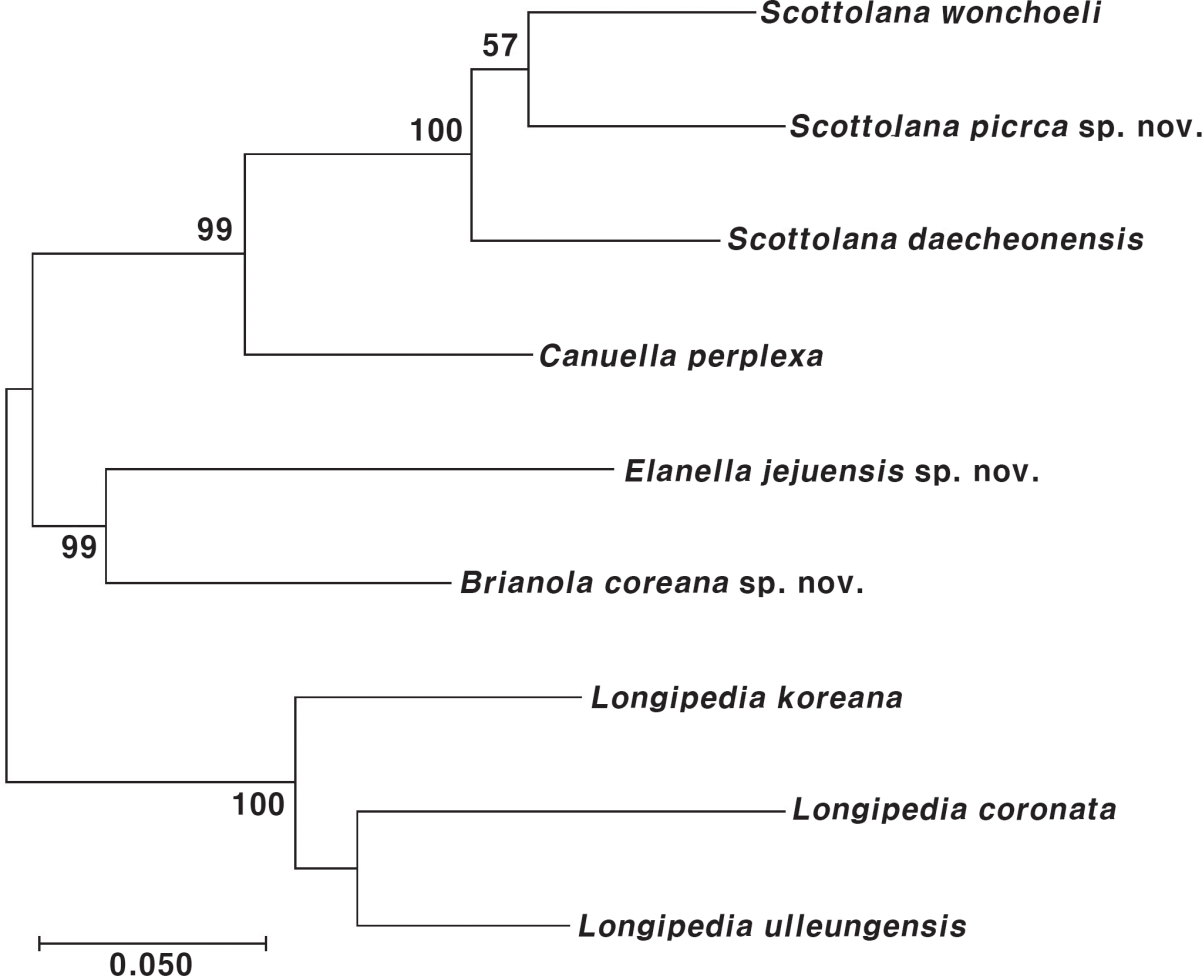
Maximum likelihood phylogenetic tree reconstructed from concatenated mtCOI and 18S rRNA sequences, showing relationships among four genera of Canuellidae, with Longipediidae as the outgroup. Numbers on branches indicate bootstrap support values (ML, %). The scale bar indicates the number of substitutions per site.

A concatenated alignment of mtCOI (658 bp) and 18S rRNA (1,658 bp) gene sequences was used to reconstruct a maximum likelihood (ML) phylogenetic tree (Fig. 33). The resulting phylogeny (scale bar: 0.05 substitutions/site) rooted using three *Longipedia* species (Longipediidae) as outgroups clearly resolved the included Canuellidae genera into two primary clades, both strongly supported by bootstrap values (99–100%).

The first main clade (99% bootstrap) grouped *Scottolana* and *Canuella*. Within the *Scottolana* lineage, *Scottolana picrca* sp. nov. was recovered as sister to *S. wonchoeli* (but with weak bootstrap support; 57%), and these two species together were strongly supported as sister to *S. daecheonensis* (100% bootstrap). The second main clade (99% bootstrap) comprised *Elanella jejuensis* sp. nov. and *Brianola coreana* sp. nov. as sister taxa.

All *Longipedia* species, used as the outgroup, were recovered as a distinct, well-supported monophyletic lineage (100%).

## Discussion

### Taxonomic affinities of the new species

*Elanella jejuensis* sp. nov.

*Elanella jejuensis* sp. nov. is assigned to the genus *Elanella* Por, 1984, based on the combination of diagnostic characters defined by Por (1984) and subsequently emended by Drumm et al. (2015). These diagnostic features include a free first pedigerous somite, a large 7-segmented antennal exopodite, P1–P3 endopods longer than their corresponding exopods, an apophysis on the male P2 enp-1 segment, and a relatively reduced male P6.

This species can be distinguished from the three known congeners (*Elanella elanitica* (Por, 1967), *E. paenelanitica* (Fiers, 1982), and *E. haifensis* Drumm, Neubert & Lubinevsky, 2015 ) by a combination of morphological characters: i) antennule segmentation is diagnostic among congeners: in females, the antennule is 4-segmented (vs 5 in *E. elanitica* and *E. paenelanitica*, and 3 in *E. haifensis*); in males, the antennule is 4-segmented in the new species, whereas it is reported as 9-segmented in *E. elanitica* and 5-segmented in both *E. paenelanitica* and *E. haifensis*; ii) the antenna differs in having a 3-segmented endopod (vs 2-segmented in congeners), and the mandible differs in having a 2-segmented exopod (vs 3-segmented in congeners); iii) the maxilliped morphology further distinguishes *E. jejuensis* sp. nov. from the other congeners: in maxilliped setation, it differs from *E. haifensis*, with the proximal segment (syncoxa) bearing nine elements (vs six in *E. haifensis*) and the basis bearing two setae (vs three in *E. haifensis*). The maxilliped also contrasts with the reduced bi-articulate condition described for *E. elanitica* and with the fully articulated condition reported for *E. paenelanitica*, in which the precoxa, coxa, basis, and endopod are all distinctly separated.

Further differences are found in swimming leg armature: P1 enp-3 bears six setae (as in *Elanella paenelanitica* and *E. haifensis*, but different from *E. elanitica*, which bears five setae), and the P4 coxa carries an inner seta in all except *E. paenelanitica*, where the seta is absent. Females of the new species are also larger (2.24 mm) than those reported for congeners (1.45–1.70 mm).

Male secondary sexual characters provide additional corroboration: the male P2 enp-1 bears a distinctive sagittate process on the outer distal corner (the male P2 endopod of *Elanella paenelanitica* is unknown), and the male P6 is a complex structure with an articulated pinnate element, contrasting with the relatively simple condition described for *E. elanitica* and *E. haifensis*.

The female caudal ramal setal modifications were emphasized as key diagnostic traits by Drumm et al. (2015); however, their diagnostic value requires careful interpretation because the setal numbering reported in Drumm et al. (2015: Fig. 2B) is not fully consistent with setal homology. Moreover, details of caudal setae IV and V are omitted in Por’s (1967) figure of *Elanella elanitica*, and a bulbous, basally spinulose terminal caudal seta is reported for *E. paenelanitica* by Fiers (1982). Accordingly, this character has limited diagnostic value when treated in isolation.

#### *Brianola coreana* sp. nov.

*Brianola coreana* sp. nov. is assigned to the genus *Brianola* Monard, 1926, based on key generic characters (Hamond 1973; Nazari et al. 2018). These include: the first pedigerous somite being fused to the cephalosome; the characteristic bell-shaped rostrum; the well-developed pseudoperculum with marginal denticulation; the reduced armature pattern of the swimming legs (P1–P4 exp-3 with 5, 4, 4, 4 elements; P2–P4 enp-3 with 5, 4, 4 elements); and swimming legs without sexual dimorphism.

The new species is easily distinguished from all known congeners by the segmentation of its cephalic appendages. It is characterized by a 3-segmented mandibular exopod. This state differs from all other species, which possess either a 1-segmented (*Brianola curvirostris* Bozic, 1968; *B. elegans* Hamond, 1973; *B. haliensis* Nazari, Mirshamsi, Sari, Aliabadian & Martínez Arbizu, 2018; *B. rawaiensis* Chullasorn, Kangtia, Klangsin & Song, 2024) or 2-segmented (*B. exigua* Por, 1967; *B. hamondi* Wells & Rao, 1987; *B. sydneyensis* Hamond, 1973; *B. vangoethemi* Fiers, 1982) exopod. Furthermore, the antennary exopod of *B. coreana* sp. nov. is 8-segmented, a character shared only with *B. haliensis* and *B. rawaiensis*. This feature separates *B. coreana* sp. nov. from *B. vangoethemi* and *B. curvirostris* (both with 7-segmented antennary exopod), and from *B. elegans*, *B. exigua*, *B. hamondi*, *B. stebleri* (Monard, 1926), and *B. sydneyensis* (all 6-segmented). In addition, the 4-segmented female antennule is also shared with *B. haliensis* and *B. rawaiensis*, differing from the 5-segmented condition found in *B. curvirostris*, *B. elegans*, *B. exigua*, and *B. stebleri*.

The swimming leg armature provides further diagnostic characters. The presence of an inner seta on the P1 coxa allies *Brianola coreana* sp. nov. with *B. haliensis*, *B. hamondi*, *B. rawaiensis*, *B. stebleri*, and *B. sydneyensis*. This clearly separates it from *B. curvirostris*, *B. elegans*, and *B. exigua*, which all lack this element.

Although *Brianola coreana* sp. nov. shares several key features with *B. haliensis* (8-segmented A2 exopod, 4-segmented A1, P1 coxa seta), the new species is readily differentiated by the presence of an inner seta on P1 exp-2; this seta is absent in *B. haliensis* and *B. exigua*.

The setation of the swimming legs distinguishes *Brianola coreana* sp. nov. from various congeners. It is armed with five elements on the P1 exp-3, differing from *B. curvirostris* and *B. elegans* (six elements), and *B. exigua* and *B. hamondi* (four elements). The P1 enp-3 bears six elements, unlike *B. stebleri* (four elements). The P2 enp-3 has five elements, separating it from *B. exigua* (four elements). Finally, the P4 enp-1 bears an inner element, which distinguishes it from *B. vangoethemi* (where this element is absent).

The male of *Brianola coreana* sp. nov. is characterized by a 5-segmented antennule and a distinct P6. The male P6 bears one bipinnate seta, three strong spinules, and an arrowhead-like element, which differs from the genital fields illustrated for other known species.

#### *Scottolana picrca* sp. nov.

Based on the recent revision by Gómez et al. (2024), the genus *Scottolana* is split into two primary assemblages based on the urosome segmentation, which are further subdivided into six species groups based on the armature of the female caudal rami. We also follow the classification proposed by Gómez et al. (2024), which recognizes two main complexes and six species groups within *Scottolana*: the first assemblage, the *longipes*-complex, is defined by the synapomorphic condition of the reduced urosome, featuring two postgenital somites in the female and three in the male. This complex is further divided into three groups (*sensu* Gómez et al., 2024): (1) the *longipes*-group (Mu & Huys, 2004), as redefined by Gómez et al. (2024), characterized by the modification of female caudal seta II, which is short and bare, with a bulbiform base, comprising *S. longipes* (Thompson I. C. & Scott A., 1903), *S. longipes sensu*Por (1964), *S. dissimilis* (Fiers, 1982), *S. wonchoeli* Bang, Moon & Back, 2022, and *S. uxoris* (Por, 1983); (2) the *brevifurca*-group (Gómez et al., 2024), defined by a “chitinous knob” modification of female caudal seta V, comprising *S. brevifurca* (Wells, 1967) and *S. gomezi* Nazari, Mirshamsi, Sari, Aliabadian & Martínez Arbizu, 2018; and (3) the *geei*-group (Gómez et al., 2024), which retains an unmodified female caudal seta II, comprising *S. geei* Mu & Huys, 2004, *S. jasani* Song, Kangtia, Khim & Chullasorn, 2018, *S. daecheonensis* Bang, Moon & Back, 2022, *S. huysi* Song, Kangtia, Khim & Chullasorn, 2018, *S. longipes sensu*Wells (1967), and *S. longipes sensu* Wells & Rao (1987). The second assemblage includes all species outside this complex, which retain the plesiomorphic 6-segmented urosome. Following Gómez et al. (2024), these are further divided into three groups: (1) the *glabra*-group (*S. glabra* Fiers, 1982, *S. oleosa* Wells & Rao, 1987, *S. rostrata* Wells & Rao, 1987), characterized by unmodified caudal setae; (2) the *bulbosa*-group, including *S. bulbosa* (Por, 1964), *S. bulbifera* (Chislenko, 1971), *S. tumidiseta* Wells & Rao, 1987, defined by a strongly bulbous base on female caudal seta II; and (3) the *antillensis*-group, including *S. antillensis* Fiers, 1984, *S. tama* Gómez, Yáñez-Rivera, García-Vázquez & Armenteros, 2024, defined by female caudal seta II being transformed into a strong, blunt, inner spine.

Following this classification, *Scottolana picrca* sp. nov. is assigned to the *longipes*-complex based on its reduced urosome. Within this complex, the new species belongs to the *longipes*-group (Mu & Huys, 2004; *sensu* Gómez et al., 2024), which is characterized by the modification of female caudal seta II. *S. picrca* sp. nov. conforms to this diagnosis, as its female caudal seta II is modified with a bulbiform base, while seta V remains unmodified and is the longest caudal seta. Accordingly, the new species is assigned to the *longipes*-complex, which currently includes *S. dissimilis*, *S. longipes*, *S. uxoris*, and *S. wonchoeli*.

*Scottolana picrca* sp. nov. can be distinguished from all other members of the *longipes*-group by the following combination of characters. It differs from *S. dissimilis* for the presence of an inner seta on P2 enp-1 (absent in *S. dissimilis*), its 3-segmented female antennule (vs 6-segmented in *S. dissimilis*), and its caudal rami, which are about 2.5 times as long as wide (vs ~3.5 times in *S. dissimilis*). The new species is separated from *S. uxoris* by the presence of the large apophysis on the P2 enp-1 and the modified pore on the male P3 enp-3, both of which are absent in *S. uxoris*. Additionally, the caudal rami of *S. picrca*, being about 2.5 times as long as wide, are much more elongate than the ‘nearly equilateral’ rami of *S. uxoris*. It differs from *S. longipes* primarily in the segmentation of the female antennule, which is 3-segmented in the new species but 7-segmented in *S. longipes*. The caudal rami proportions of *S. picrca* (about 2.5 times as long as wide) fall within the range reported for *S. longipes* (2.0 to 3.0).

*Scottolana picrca* sp. nov. shares several diagnostic features with *S. wonchoeli*, including a 3-segmented female antennule, the presence of an apophysis on P2 enp-1, a modified pore on the male P3 enp-3, and comparable caudal rami proportions (about 2.5 times as long as wide in the new species vs ~2.6 in *S. wonchoeli*). Nevertheless, *S. picrca* sp. nov. can be readily distinguished from *S. wonchoeli* by the following combination of characters: 1) a proportionally longer rostrum (rostrum/cephalic shield ratio ~0.61 in *S. picrca* sp. nov., ~0.45 in *S. wonchoeli*), markedly smaller body length (908 and 1,820 µm, respectively), and a slightly lower body length/width ratio (4.05 and 4.44, respectively); 2) female caudal seta II short, bare, and basally bulbiform in the new species, but small and conical in *S. wonchoeli*; 3) the male antennule 3-segmented in the new species, but 5-segmented in *S. wonchoeli*; and 4) differences in swimming leg armature, including serrate outer spines on P1 enp-3, a bipinnate spine as the inner element on P3 enp-1, and plumose inner setae on P4 exp-2 and P4 enp-1 (the corresponding elements pinnate and/or setiform in *S. wonchoeli*). Finally, the male genital field differs from that of *S. wonchoeli*, most notably by the much longer internal process arising near the proximal inner margin of P6 (extremely elongate and barbed in *S. picrca* sp. nov.) and by the P6 process bearing a bare inner seta subapically in the new species, whereas the corresponding seta is pinnate in *S. wonchoeli*.

The new species is readily excluded from the *brevifurca*-group, which includes species such as *Scottolana gomezi*, as this group is defined by the modification of female caudal seta V; in *S. picrca* sp. nov., caudal seta V is unmodified. Further distinctions from *S. gomezi* include: (1) the 3-segmented mandibular exopod (vs imperfectly separated in *S. gomezi*); and (2) the presence of a large, modified pore on the male P3 enp-3, a feature explicitly noted as absent in *S. gomezi*. Finally, *S. picrca* sp. nov. is excluded from the *geei*-group, which includes many of the “Asian complex” species such as *S. geei*, *S. huysi*, *S. jasani*, and *S. daecheonensis*. This group is characterized by an unmodified female caudal seta II, whereas seta II is modified in the new species.

### Phylogenetic relationships

The phylogenetic analysis, based on concatenated mitochondrial COI and nuclear 18S rRNA gene sequences, presents molecular evidence (99% bootstrap support) for a sister-group relationship between the genera *Elanella* and *Brianola*. This discovery holds taxonomic importance, as morphological data alone had not previously suggested such a close relationship. *Brianola* is defined by the apomorphic fusion of the first pedigerous somite to the cephalosome, whereas *Elanella* retains the plesiomorphic free somite. Their highly supported phylogenetic linkage suggests this fusion event is a critical apomorphy for *Brianola*, and that *Elanella* may represent the sister lineage retaining the plesiomorphic condition. Together, they form a well-supported clade distinct from the *Scottolana* + *Canuella* clade (99% bootstrap), further reinforcing the deep phylogenetic divisions within the family.

Within the genus *Scottolana*, our analysis tentatively places *S. picrca* sp. nov. (from Palau) as sister to *S. wonchoeli* (from Korea), but with weak support (57%). This placement is consistent with their shared morphological diagnoses (e.g., reduced urosome, modified female caudal seta II) that place them in the *longipes*-group (*sensu* Gómez et al., 2024). However, the low bootstrap support at this specific node (57%) highlights a key limitation, which is explored further below.

### Limitations and future perspectives

Biogeographically (Bang et al. 2022), the *longipes*-group of *Scottolana* displays a broadly scattered yet coherent distribution across major marine regions, including the Indo-West Pacific region, encompassing the Indian Ocean (with *S. longipes* from the Gulf of Manaar), the western and southwestern Pacific (including *S. dissimilis* from Papua New Guinea and *S. picrca* sp. nov. from Palau), the northwestern Pacific represented by the East Sea (*S. wonchoeli* from Korea), and the transitional zone between the western Indo-Pacific and Mediterranean, including the Red Sea and Mediterranean basin (with *S. longipes sensu* Por from the Mediterranean coast of Israel and *S. uxoris* from the Gulf of Elat). Such regional grouping reflects marine biogeographic provinces rather than isolated localities, highlighting connectivity through oceanographic features and potential dispersal corridors. The considerable geographic gaps between these marine provinces imply under-sampling in intermediate areas such as Southeast Asia and Micronesia. Furthermore, records of habitat depth and environmental conditions can provide critical information. To fully elucidate the evolutionary history and contemporary dispersal of this group, expanded sampling efforts particularly targeting these marine transition zones are warranted.

Within the family Canuellidae, partial mitochondrial COI and 18S rRNA gene sequences are available for only four of the 18 described genera (see Walter and Boxshall 2026), which imposes limitations on conducting a comprehensive phylogenetic analysis. This limitation is most evident in the low bootstrap support (57%) for the node placing *Scottolana picrca* sp. nov. as sister to *S. wonchoeli* (Fig. 33). Additionally, the distinct position of *S. daecheonensis* suggests a complex history within the Asian *Scottolana* lineage that warrants deeper investigation. Resolving ambiguous phylogenetic nodes and constructing a comprehensive tree clearly requires broader taxon sampling and additional loci, ideally phylogenomic data. The current practice of omitting genetic data in many new species descriptions (Bouchet et al. 2023) is a significant limitation. For taxonomically challenging groups such as canuellids, where species identification relies on complex appendage morphology demanding expert knowledge, the provision of genetic data for rapid identification is essential. Accordingly, integrative taxonomy that combines expanded molecular datasets with detailed morphological re-examination will be critical. Such an integrative framework will not only clarify the evolutionary history of the Canuellidae but also enable the rapid identification of canuellids, establishing a fundamental baseline for actively investigating copepod biodiversity in the Western Pacific.

### Updated identification key to the species of *Scottolana* Huys, 2009

The genus *Scottolana* has seen significant taxonomic additions and revisions since its establishment. The identification key provided below amends the previous key published by Bang et al. (2022) by adopting the revised classification framework recently proposed by Gómez et al. (2024). This framework organizes the genus into complexes and species groups based primarily on urosome segmentation and female caudal armature. The key incorporates the new species described herein (*S. picrca* sp. nov.).

Notes: **^†^***S. longipes sensu*Por (1964) also lacks the modified pore on the male P3 enp-3, leading it to key out at the same couplet (13) as *S. uxoris*. However, these two taxa can be distinguished: *S. longipes sensu*Por (1964) has a 5-segmented female antennule and caudal seta II located in the proximal third of the ramus, whereas *S. uxoris* (male unknown for P3 pore character confirmation, female antennule segmentation unknown) has nearly equilateral caudal rami and lacks the P2 enp-1 apophysis found in other *longipes*-group members.

**Table:** 

1	Female urosome with 2 postgenital somites (male with 3); P4 often modified	**(*longipes* complex) 2**
–	Female urosome with 3 postgenital somites (male with 4); P4 not modified as above	**(plesiomorphic state) 14**
2	Female caudal seta V modified, transformed into a “chitinous knob”	**(*brevifurca* group) 3**
–	Female caudal seta V unmodified, setiform	**4**
3	Mandibular exopod segments well-separated	***Scottolana brevifurca***
–	Mandibular exopod segments imperfectly separated	***S. gomezi***
4	Female caudal seta II setiform, typically long and pinnate (unmodified)	**(*geei* group) 5**
–	Female caudal seta II distinctly shortened, bare, often with bulbous base (modified)	**(*longipes* group) 9**
5	Female caudal seta II with distinct bulbous base	***S. huysi***
–	Female caudal seta II with normal base	**6**
6	Antennary exopod 9-segmented	***S. geei***
–	Antennary exopod 8-segmented	**7**
7	Male P3 enp-3 without modified tube-pore; female antennule 4-segmented	***S. daecheonensis***
–	Male P3 enp-3 with modified tube-pore; female antennule 3-segmented	**8**
8	P2–P4 basis without distinct inner processes	***S. jasani***
–	P2–P4 basis with distinct inner processes	***S. longipes sensu*Wells (1967) / Wells & Rao (1987)**
9	Female antennule 3-segmented	**10**
–	Female antennule with 5 or more segments	**11**
10	Female caudal seta II small and conical; male antennule 5-segmented	***S. wonchoeli***
–	Female caudal seta II short, bare, with bulbiform base; male antennule 3-segmented	***S. picrca* sp. nov**.
11	Female antennule 6-segmented	***S. dissimilis***
–	Female antennule 5- or 7-segmented	**12**
12	Female antennule 5-segmented; caudal seta II located in proximal third of ramus	***S. longipes sensu* Por (1964) ^†^**
–	Female antennule 7-segmented; caudal seta II located about halfway along ramus	**13**
13	Male P3 enp-3 with modified pore	***S. longipes***
–	Male P3 enp-3 without modified pore	***S. uxoris***
14	Female caudal seta II transformed into a strong, blunt inner spine	**(*antillensis* group) 15**
–	Female caudal seta II not a strong, blunt spine	**16**
15	Mandibular exp-1 with 2 setae; maxillulary exopod with 11 setae	***S. tama***
–	Mandibular exp-1 with 1 seta; maxillulary exopod with 10 setae	***S. antillensis***
16	Female caudal seta II strongly bulbous with whip-like distal part	**(*bulbosa* group) 17**
–	Female caudal seta II unmodified, long and setose (or not strongly bulbous)	**19**
17	P2 enp-3 with 6 setae; P4 exp-3 with 5 setae	***S. bulbosa***
–	P2 enp-3 with 5 setae; P4 exp-3 with 4 setae	**18**
18	Antennary exopod 8-segmented	***S. bulbifera***
–	Antennary exopod 7-segmented	***S. tumidiseta***
19	P1 enp-1 without inner seta	***S. scotti*** ^*^
–	P1 enp-1 with inner seta	**20**
20	Caudal seta II long and smooth (not pinnate)	*S. inopinata* ^*^
–	Caudal seta II pinnate (as normal)	**(*glabra* group) 21**
21	Caudal ramus about as long as wide	***S. glabra***
–	Caudal ramus longer than wide	**22**
22	Caudal ramus about 1.5 times as long as wide	***S. rostrata***
–	Caudal ramus about 2 times as long as wide	***S. oleosa***

## Supplementary Material

XML Treatment for Elanella jejuensis

XML Treatment for Brianola coreana

XML Treatment for Scottolana picrca

## Footnotes

Considered incertae sedis within Scottolana by Gómez et al. (2024) due to an insufficient description.

## Additional information

### Use of AI

No use of AI was reported.

### Author ORCIDs

Hyun Woo Bang https://orcid.org/0000-0002-9626-9762

Jinwook Back https://orcid.org/0000-0003-0285-1135

### Data availability

The sequence data generated in this study are available in the GenBank repository under accession numbers PP297485–PP297491 (mtCOI) and PP298071–PP298077 (18S rRNA). Type specimens are deposited in the National Marine Biodiversity Institute of Korea (MABIK) under the accession numbers detailed in the ‘Type material’ sections (MABIK CR00259503–CR00259512; MABIK CR00242447–CR00242534). All other morphological data are available within the article.

## References

[B1] Bang HW, Moon H, Back J (2022) Two new species of the family Canuellidae Lang, 1944 (Copepoda: Polyarthra), from Korea, with a key to species of the genus *Scottolana* Huys, 2009. Diversity 14(11): 967. 10.3390/d14110967

[B2] Bernot JP, Khodami S, Boyen J, De Troch M, Boxshall GA, Martínez Arbizu P (2025) Copepod phylogenomics supports Canuelloida as a valid order separate from Harpacticoida. Molecular Phylogenetics and Evolution 206: 108311. 10.1016/j.ympev.2025.108311 39986405

[B3] Bouchet P, Decock W, Lonneville B, Vanhoorne B, Vandepitte L (2023) Marine biodiversity discovery: The metrics of new species descriptions. Frontiers in Marine Science 10: 929989. 10.3389/fmars.2023.929989

[B4] Boxshall GA, Halsey SH (2004) An Introduction to Copepod Diversity. The Ray Society, London, 966 pp.

[B5] Bozic B (1968) Copépodes de La Réunion. III. *Brianola curvirostris* n. sp. Bulletin du Muséum National d’Histoire Naturelle, Paris, 2e série 40(3): 570–573.

[B6] Chislenko LL (1971) Novye massovye formy garpaktitsid (Copepoda, Harpacticoida) iz zaliva Pos’eta Yaponskogo morya. Issledovaniya Fauny Morei 8(16): 151–181. [New common forms of harpacticids (Copepoda, Harpacticoida) from Possjet Bay of the Sea of Japan] [In Russian]

[B7] Chullasorn S, Kangtia P, Klangsin P, Song SJ (2024) A new species of *Brianola* Monard, 1926 (Copepoda: Harpacticoida: Canuellidae) from Rawai Beach, Phuket Island, Thailand. Journal of Species Research 13(3): 340–351. 10.12651/JSR.2024.13.3.340

[B8] Coull BC (1999) Role of meiofauna in estuary ecosystems. Australian Journal of Ecology 24(4): 327–343.

[B9] Coull BC, Chandler GT (1992) Pollution and meiofauna: Field, laboratory, and mesocosm studies. Oceanography and Marine Biology - an Annual Review 30: 191–271.

[B10] Dahms H-U (1990) Naupliar development of Harpacticoida (Crustacea, Copepoda) and its significance for phylogenetic systematics. Meiofauna Marina 6: 169–272.

[B11] Dahms H-U (2004a) Exclusion of the *Polyarthra* from Harpacticoida and its reallocation as an underived branch of the Copepoda (Arthropoda, Crustacea). Zoologia Bespozvonocnyh 1(1): 29–51. 10.15298/invertzool.01.1.03

[B12] Dahms H-U (2004b) Postembryonic apomorphies proving the monophyletic status of the Copepoda. Zoological Studies (Taipei, Taiwan) 43(2): 446–453.

[B13] Drumm DT, Neubert PL, Lubinevsky H (2015) A new species of the genus *Elanella* (Copepoda: Harpacticoida: Canuellidae) from the eastern Mediterranean Sea. Proceedings of the Biological Society of Washington 128(1): 40–50. 10.2988/0006-324X-128.1.40

[B14] Fiers F (1982) New Canuellidae from the northern coast of Papua New Guinea (Copepoda: Harpacticoida). Bulletin de l’Institut Royal des Sciences Naturelles de Belgique. Biologie 54(4): 1–32.

[B15] Giere O (2009) Meiobenthology: The Microscopic Fauna in Aquatic Sediments (2^nd^ Ed.). Springer-Verlag, Berlin Heidelberg, 527 pp.

[B16] Gómez S, Yáñez-Rivera B, García-Vázquez L, Armenteros M (2024) On some new species of Canuelloida Khodami, Vaun MacArthur, Blanco-Bercial & Martinez Arbizu, 2017 (Crustacea: Copepoda) from a shallow coastal lagoon in north-western Mexico. Zootaxa 5555(4): 497–534. 10.11646/zootaxa.5555.4.2 40174038

[B17] Hamond R (1973) Four new copepods (Crustacea: Harpacticoida, Canuellidae) simultaneously occurring with *Diogenes senex* (Crustacea: Paguridae) near Sydney. Proceedings of the Linnean Society of New South Wales 97(3): 165–201.

[B18] Heip C, Vincx M, Vranken G (1985) The ecology of marine nematodes. Oceanography and Marine Biology - an Annual Review 23: 399–489.

[B19] Hicks GRF, Coull BC (1983) The ecology of marine meiobenthic harpacticoid copepods. Oceanography and Marine Biology - an Annual Review 21: 67–175.

[B20] Huys R, Boxshall GA (1991) Copepod Evolution. The Ray Society, London, 468 pp.

[B21] Huys R, Gee JM, Moore CG, Hamond R (1996) Marine and Brackish Water Harpacticoid Copepods Part 1. Synopses of the British Fauna 51: 1–352. [New Series]

[B22] ICZN [International Commission on Zoological Nomenclature] (1999) International Code of Zoological Nomenclature. Fourth Edition. Ride WDL, Cogger HG, Dupuis C, Kraus O, Minelli A, Thompson FC, Tubbs PK (Eds) The International Trust for Zoological Nomenclature, London, 306 pp. https://www.iczn.org/the-code/the-code-online

[B23] ICZN [International Commission on Zoological Nomenclature] (2023) Declaration 46. Amendment of Article 8.8. International Code of Zoological Nomenclature. The Bulletin of Zoological Nomenclature 80(1): 6–7. 10.21805/bzn.v80.a002

[B24] Katoh K, Standley DM (2013) MAFFT Multiple Sequence Alignment Software Version 7: Improvements in Performance and Usability. Molecular Biology and Evolution 30(4): 772–780. 10.1093/molbev/mst010 PMC3603318 23329690

[B25] Kearse M, Moir R, Wilson A, Stones-Havas S, Cheung M, Sturrock S, Buxton S, Cooper A, Markowitz S, Duran C, Thierer T, Ashton B, Meintjes P, Drummond A (2012) Geneious Basic: An integrated and extendable desktop software platform for the organization and analysis of sequence data. Bioinformatics (Oxford, England) 28(12): 1647–1649. 10.1093/bioinformatics/bts199 PMC3371832 22543367

[B26] Khodami S, McArthur JV, Blanco-Bercial L, Martinez Arbizu P (2017) Molecular phylogeny and revision of copepod orders (Crustacea: Copepoda). Scientific Reports 7(1): 9164. 10.1038/s41598-017-06656-4 PMC5567239 28831035

[B27] Khodami S, McArthur JV, Blanco-Bercial L, Martinez Arbizu P (2020) Retraction Note: Molecular phylogeny and revision of copepod orders (Crustacea: Copepoda). Scientific Reports 10(1): 17743. 10.1038/s41598-020-74404-2 PMC7560885 33057148

[B28] Kimura M (1980) A simple method for estimating evolutionary rates of base substitutions through comparative studies of nucleotide sequences. Journal of Molecular Evolution 16(2): 111–120. 10.1007/BF01731581 7463489

[B29] Lang K (1948) Monographie der Harpacticiden. Håkan Ohlsson, Lund, 1682 pp.

[B30] Lim BJ, Bang HW, Moon H, Back J (2020) Integrative description of *Diosaccus koreanus* sp. nov. (Hexanauplia, Harpacticoida, Miraciidae) and integrative information on further Korean species. ZooKeys 927: 1–35. 10.3897/zookeys.927.49042 PMC7180166 32341672

[B31] Monard A (1926) Sur les Harpacticus de Banyuls. Bulletin de la Société Zoologique de France 51: 419–434.

[B32] Mu F-H, Huys R (2004) Canuellidae (Copepoda, Harpacticoida) from the Bohai Sea, China. Journal of Natural History 38(1): 1–36. 10.1080/00222930210138935

[B33] Nazari F, Mirshamsi O, Sari A, Aliabadian M, Martínez Arbizu P (2018) Three new Canuellidae (Copepoda: Canuelloida) from Iran. Zootaxa 4446(4): 401–441. 10.11646/zootaxa.4446.4.1 30313868

[B34] Nei M, Kumar S (2000) Molecular Evolution and Phylogenetics. Oxford University Press, New York, 333 pp.

[B35] Por FD (1964) A study of the levantine and pontic harpacticoida (Crustacea, Copepoda). Zoologische Verhandelingen, Leiden 64: 1–128.

[B36] Por FD (1967) Level bottom Harpacticoida (Crustacea, Copepoda) from Elat (Red Sea), part I. Israel Journal of Zoology 16: 101–165.

[B37] Por FD (1983) A note on two new species of Canuellidae (Copepoda, Harpacticoida) from the Red Sea. Crustaceana 44(2): 187–197. 10.1163/156854083X00802

[B38] Por FD (1984) Canuellidae Lang (Harpacticoida, Polyarthra) and the ancestry of the Copepoda. Crustaceana (Supplement 7): 1–24. 10.1163/9789004629363_003

[B39] Song SJ, Kangtia P, Khim JS, Chullasorn S (2018) Two new Asian species of the genus *Scottolana* Huys, 2009 (Copepoda: Canuelloida: Canuellidae). Journal of Natural History 52(7–8): 377–403. 10.1080/00222933.2018.1432777

[B40] Thompson IC, Scott A (1903) Report on the Copepoda collected by Professor Herdman, at Ceylon, in 1902. Report to the Government of Ceylon on the Pearl Oyster Fisheries of the Gulf of Manaar 1(Supplement 7): 227–307. 10.5962/bhl.title.59334

[B41] Tiemann H (1984) Is the taxon Harpacticoida monophyletic? Crustaceana (Supplement 7): 47–59. 10.1163/9789004629363_006

[B42] Vakati V, Dodsworth S (2020) Non-destructive genome skimming for aquatic copepods. Conservation Genetics Resources 12(3): 515–520. 10.1007/s12686-020-01129-9

[B43] Walter TC, Boxshall GA (2026) World of Copepods Database. World Register of Marine Species. https://www.marinespecies.org/copepoda [Accessed on 26.01.2026]

[B44] Wells JBJ (1967) VII. The Littoral Copepoda (Crustacea) of Inhaca Island, Mozambique. Transactions of the Royal Society of Edinburgh 67(7): 189–358. 10.1017/S0080456800024017

[B45] Wells JBJ, Rao GC (1987) Littoral Harpacticoida (Crustacea: Copepoda) from Andaman and Nicobar Islands. Memoirs of the Zoological Survey of India 16(4): 1–385.

